# The Optics and Alignment of the Divergent Beam Laboratory X-ray Powder Diffractometer and its Calibration Using NIST Standard Reference Materials

**DOI:** 10.6028/jres.120.013

**Published:** 2015-09-25

**Authors:** James P. Cline, Marcus H. Mendenhall, David Black, Donald Windover, Albert Henins

**Affiliations:** National Institute of Standards and Technology, Gaithersburg, MD 20899 USA

**Keywords:** calibration, fundamental parameters approach, instrument alignment, lattice parameters, profile fitting, Rietveld analysis, Standard Reference Material, X-ray powder diffraction

## Abstract

The laboratory X-ray powder diffractometer is one of the primary analytical tools in materials science. It is applicable to nearly any crystalline material, and with advanced data analysis methods, it can provide a wealth of information concerning sample character. Data from these machines, however, are beset by a complex aberration function that can be addressed through calibration with the use of NIST Standard Reference Materials (SRMs). Laboratory diffractometers can be set up in a range of optical geometries; considered herein are those of Bragg-Brentano divergent beam configuration using both incident and diffracted beam monochromators. We review the origin of the various aberrations affecting instruments of this geometry and the methods developed at NIST to align these machines in a first principles context. Data analysis methods are considered as being in two distinct categories: those that use empirical methods to parameterize the nature of the data for subsequent analysis, and those that use model functions to link the observation directly to a specific aspect of the experiment. We consider a multifaceted approach to instrument calibration using both the empirical and model based data analysis methods. The particular benefits of the fundamental parameters approach are reviewed.

## 1. Introduction

The laboratory X-ray powder diffractometer offers several virtues that have rendered it a principal characterization device providing critical data for a range of technical disciplines involving crystalline materials. A continuous suite of hkl reflections can be collected with a single scan in θ – 2θ angle space. The sample is typically composed of small, 5 μm to 30 μm, crystallites, a format that is amenable to a wide variety of materials. Not only can timely qualitative analyses be realized, but with the more advanced data analysis methods a wealth of quantitative information may be discerned. Modern commercial instruments may embody features that include focusing mirror optics and an ability to interchange between various experimental configurations in a timely manner. We discuss results from a NIST-built diffractometer with features specific to the collection of data that complement the NIST effort in Standard Reference Materials (SRMs) for powder diffraction. While this machine can be configured with focusing optics, herein we consider only those configurations utilizing a divergent beam in Bragg-Brentano, para-focusing geometry.

A principal advantage of the divergent beam X-ray powder diffractometer (XRPD) is that a relatively large number of crystallites are illuminated, providing a strong diffraction signal from a representative portion of the sample. However, the para-focusing optics of laboratory diffractometers produce patterns that display profiles of a most complex shape. The observed 2θ position of maximum diffraction intensity does not necessarily reflect the true spacing of the hkl planes. While advanced data analysis methods can be used to model the various aberrations and account for the observed profile shape and position, there are a number of instrumental effects about which knowledge is insufficient for reliable, *a priori* modeling of instrument performance. The task may be further compounded when instruments are set up incorrectly, because the resultant additional errors are convoluted into the already complex aberration set. Therefore, the results are often confounding, as the origin of difficulty is problematic to discern. The preferred method to avoid these situations is the use of SRMs to calibrate instrument performance. We describe the various methods wherein NIST SRMs may be used to determine sources of measurement error as well as the procedures to properly calibrate the laboratory X-ray diffraction instrument.

The software discussed throughout this manuscript will include commercial as well as public domain programs, some of which were used for the certification of NIST SRMs. In addition to the NIST disclaimer concerning use of commercially available resources^1^, we emphasize that some of the software presented herein was also developed to a certain extent through longstanding collaborative relationships between the first author and the respective developers of the codes. The codes to be discussed include: *GSAS* [1], the PANalytical software *HighScore Plus* [2], the Bruker codes *TOPAS* and *EVA* [3], and the Rigaku code *PDXL 2* [4]. The fundamental parameters approach (FPA), Cheary and Coelho [5], method for the modeling of X-ray powder diffraction line profiles, as implemented in *TOPAS*, has been used since the late 1990’s for the certification of NIST SRMs. To examine the efficacy of the FPA models, as well as their implementation in *TOPAS*, we have developed a Python-based code, the NIST Fundamental Parameters Approach Python code (*FPAPC*), that replicates the FPA method in the computation of X-ray powder diffraction line profiles, Mendenhall *et al.* [6]. This FPA capability is to be incorporated into *GSAS II*, Toby and Von Dreele [7].

## 2. The Instrument Profile Function

**Fig. 1:**
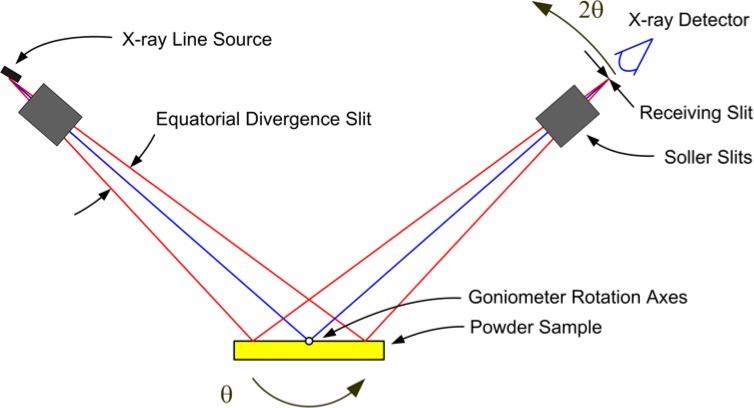
A schematic diagram illustrating the operation and optical components of a Bragg-Brentano X-ray powder diffractometer.

The instrument profile function (IPF) describes the profile shape and displacement in 2θ angle that is the intrinsic instrumental response imparted to any data collected with the specific instrument. It is a function of the radiation used, instrument geometry and configuration, slit sizes, *etc*. The basic optical layout of a divergent beam X-ray powder diffractometer of Bragg-Brentano, para-focusing geometry, utilizing the tube anode in a line source configuration is illustrated in Fig. 1. This figure portrays the various optical components in the plane of diffraction, or equatorial plane. It is the dimensions of the optical components shown, and those of the goniometer itself that determine the resolution of the diffractometer. The divergent nature of the X-ray beam will increase the number of crystallites giving rise to the diffraction signal; the incident beam slit defines an angular range within which crystallites will be oriented such that their diffraction is registered. One of the manifestations of this geometry is that knowledge of both the diffraction angle and sample position are critical for the correct interpretation of the data. The goniometer radius is the distance between the rotation axes and the X-ray source, or receiving slit as shown in Fig. 1; these two distances must be equal. The sample surface is presumed to be on the rotation axes; however this condition is rarely realized, it is common to consider a sample displacement error.

Goniometer assemblies themselves can be setup in several configurations. Figure 1 illustrates a machine of “theta/two-theta” geometry: the tube is stationary while the sample rotates through angle θ and the detector rotates through angle 2θ. Another popular configuration is “theta/theta” geometry wherein the sample remains stationary and both the tube and detector rotate through angle θ. The diffraction optics themselves do not vary with regards to how the goniometer is setup, however.

The detector illustrated in Fig. 1 simply reads any photons arriving at its entrance window, as the diffracted signal is “analyzed” by the receiving slit. Such detectors, often utilizing a scintillation crystal, are typically referred to as “point” detectors. To filter the scattered radiation of any fluorescence from the sample, a diffracted-beam, post (sample) monochromator is often appended onto the beam path after the receiving slit. The crystal optic of these monochromators typically consists of pyrolytic graphite that is bent to a radius in rough correspondence to that of the goniometer. Such an optic, with a high level of mosaicity, imposes a relatively broad energy bandpass of approximately 200 eV (with 8 keV Cu Kα) in width on the diffracted beam. This window is centered so as to straddle that of the energy of the source radiation being used, thereby filtering fluorescent and other spurious radiation from the detector while transmitting the primary features of the emission spectrum, presumably without distortion.

**Fig. 2:**
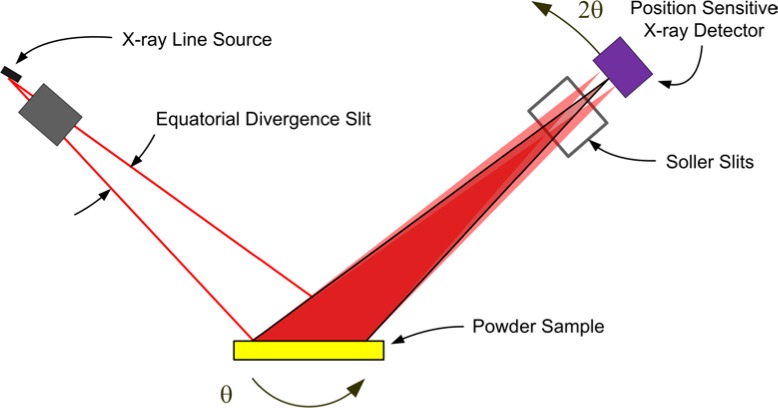
A schematic diagram illustrating the operation and optical components of a Bragg-Brentano X-ray diffractometer equipped with a position sensitive detector. Only the rays striking the centerline of the PSD, outlined in black, are in accordance to Bragg-Brentano focusing.

Within the last decade, however, the popularity of this geometry has fallen markedly, as the use of the post-monochromator/point detector assembly has been largely displaced by the use of a position sensitive detector (PSD). This geometry is illustrated in Fig. 2. A line detector replaces the point detector, and offers the ability to discriminate with respect to the position of arriving X-rays within the entrance window of the PSD. A multi-channel analyzer is typically used to map the arriving photons from the PSD window into 2θ space. Depending on the size of the PSD entrance window, increases in the counting rate, relative to a point detector, by two orders of magnitude can be easily achieved. Furthermore, this is accomplished by including the signal from additional crystallites, mitigating the difficulty of particle counting statistics. A drawback to the PSD is that the increased intensity is achieved with the inclusion of signals that are not within the Bragg-Brentano focusing regimen, Fig. 1 vs. Fig. 2, leading to a broadening of the line profiles. The level of broadening is proportional to the size of the PSD entrance window and inversely proportional to 2θ angle. The move to PSDs has been further augmented by the development of solid-state, silicon strip detectors that offer the advantages of a PSD without the maintenance issues of the early, gas-flow proportional PSDs. Fluorescence can be problematic with a PSD; however, the problem can be countered with the use of filters. More recent developments in electronics have improved the ability of these PSDs to discriminate with respect to energy. We discuss only this newer class of solid state linear PSDs herein.

**Fig. 3:**
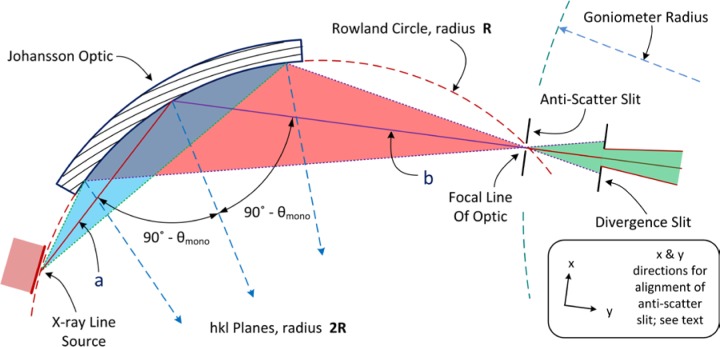
A schematic diagram illustrating the geometry of a Johansson incident beam monochromator.

A monochromator can also be used to prepare the incident beam so as to consist exclusively of Kα_1_ radiation. Monochromators of this nature are inserted into the beam path prior to the beam’s arrival at the incident beam slit portrayed in Fig. 1. These devices typically use a germanium (111) crystal as the optic, and as such exhibit a much smaller energy bandpass than graphite monochromators. They are, therefore, much more complex and difficult to align. We discuss herein an incident beam monochromator (IBM) utilizing a Johansson focusing optic [8], a diagram of which is shown in Fig. 3. With the incorporation of an IBM assembly into a powder diffractometer of reflection geometry, the focal “line” of the optic must be positioned on the goniometer radius as per the line source of the tube anode in a conventional setup, shown in the right-hand side of Fig. 3. In this way, a Johansson optic provides a “monochromatic” X-ray source, passing some portion of the Kα_1_ emission spectrum, while preserving the divergent beam Bragg-Brentano geometry as shown in Figs. 1 and 2. The use of an IBM reduces the number of contributions to the observed line shape and results in an IPF that is more readily modeled with conventional profile fitting. Furthermore, equipping such a machine with a PSD affords all of its advantages, while the elimination of the Bremsstrahlung by the IBM reduces the impact of fluorescence that can otherwise be problematic with a PSD.

Throughout this manuscript we speak of the “width” and “length” of the optics. “Width” is intended to express extent in the equatorial plane, *e.g*.; the width of the incident beam slit is 1 mm. “Length” is used to denote a physical dimension parallel to the rotation axes of the goniometer as defined in Fig. 1, *e.g.*, the length of the incident beam slit is 15 mm. The designation of the axial divergence angle, as well as the specifications concerning Soller slits, will be considered in terms of the double angle, both for incoming and outgoing rays. This is in contrast to the generally accepted single angle definition shown in Klug and Alexander [9]; as such the axial divergence angles reported throughout this manuscript will be twice those that are often encountered.

**Fig. 4:**
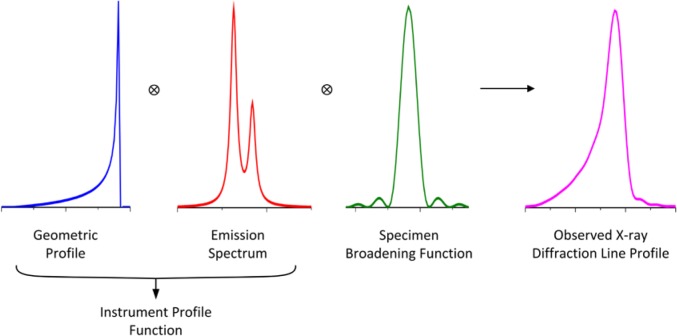
Diagrammatic representations of convolutions leading to the observed XRPD profile.

The observed line shape in powder diffraction consists of a convolution of contributions from the instrument optics, referred to as the geometric profile, the emission spectrum, and the specimen, as shown diagrammatically for divergent beam XRPD, Fig. 4. The specimen contribution is often the dominant one in a given experiment; however, we do not consider it to any great extent in this discussion. The factors comprising the geometric profile are delineated in Table 1. Technically, neither of the last two items, specimen transparency and displacement, are components of the geometric profile of the instrument. They are functions of the specimen and the manner in which it was mounted. However, it is simply impossible to use a “whole pattern” data analysis method without consideration of these two factors; they play a critical role in the modeling of the observed profile positions and shapes. Hence they are included in this discussion. The convolution of the components of the geometric profile and emission spectrum form the IPF. As will be discussed, both of these contributions are complex in nature, leading to the well-known difficulty in modeling the IPF from Bragg-Brentano equipment. This complexity, and the relatively limited q-space (momentum space) range accessible with laboratory equipment, tends to drive the structure solution and refinement community, with their expertise in the development of data analysis procedures, towards the use of synchrotron and neutron sources. A significant portion of the models and analytical functions discussed herein were developed for, and are better suited to, powder diffraction equipment utilizing such nonconventional sources.

**Fig. 5:**
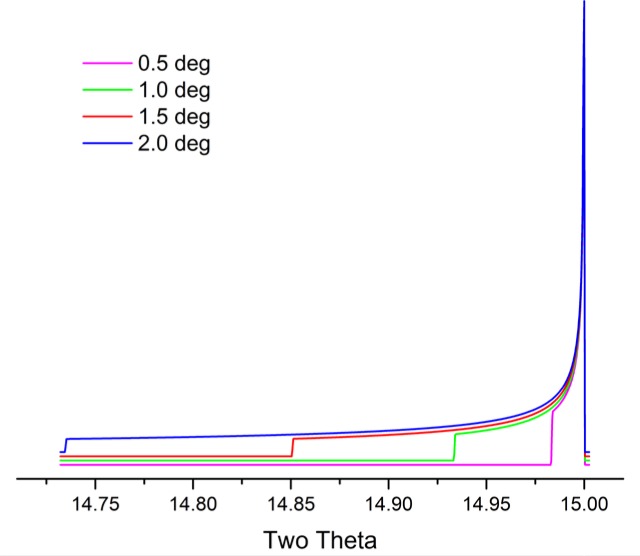
The flat specimen error aberration profile as a function of incident slit size, R = 217.5 mm.

**Fig. 6:**
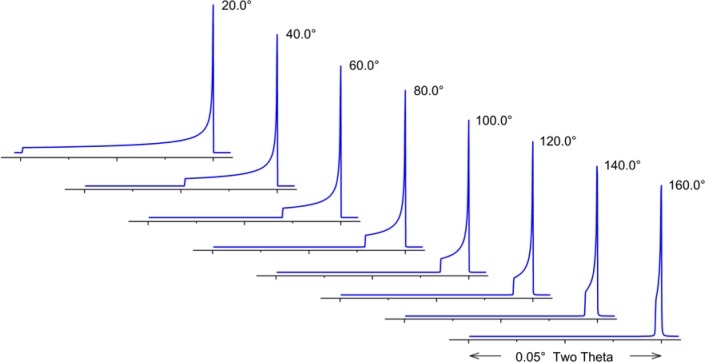
The flat specimen error aberration profiles for a 1° incident slit as a function Two-Theta, R = 217.5 mm.

We now consider the geometric profile with an examination of the aberrations listed in Table 1. Figures 5 through 10 illustrate simulations of the aberration function itself associated with the factors listed in Table 1. The first two of these, the source and receiving slit width or silicon strip width with a PSD, simply cause symmetric broadening, constant with 2θ angle, and are typically described with “impulse” or “top hat” functions. The flat specimen error is due to defocusing in the equatorial plane. One can see from inspection of Fig. 1 that for any beam that is not on the centerline of the goniometer, R_1_ will not equal R_2_. The magnitude of the effect is directly proportional to the divergent slit size as shown in Fig. 5. Its functional dependence on 2θ angle, *i.e.*, 1/tanθ, is illustrated in Fig. 6. The flat specimen error leads to asymmetric profile broadening on the low angle side, accentuated with decreasing values of 2θ. The functional dependence of this aberration on 2θ, Fig. 6, is for a fixed slit; use of a variable divergence incident beam slit to obtain constant area of illumination reduces this dependence on the 2θ angle.

**Fig. 7:**
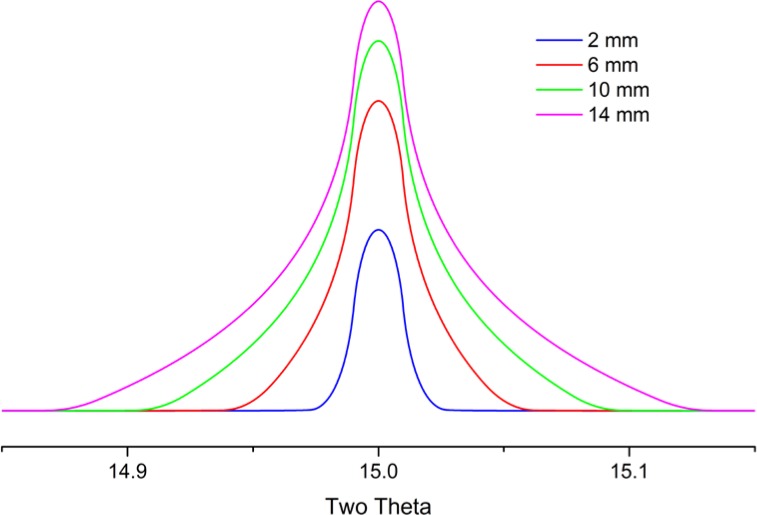
The PSD defocusing error aberration profiles for a silicon strip PSD as a function of window width, R = 217.5 mm, incident slit = 1° and the strip width = 75 μm.

The broadening imparted to diffraction line profiles from the early gas-flow proportional PSDs was due to defocusing originating from both the equatorial width of the PSD window and parallax within the gas-filled counting chamber. Early models for these effects, Cheary and Coelho [10], included two parameters, one for the window width and a second for the parallax. The modern silicon strip PSDs do not need this second term as there is effectively no parallax effect. The aberration profile imparted to the data from a modern PSD, Mendenhall, is illustrated in Fig. 7 as a function of window width. The profiles are symmetric about the centerline, exhibiting both increasing intensity and breadth as the window width is increased. The profile consists of two components: a central peak with a width independent of 2θ, which is due to the pixel strip width of the detector, and wings which are due to the defocusing. The breadths of the wings shown in Fig. 7 vary in a manner proportional to the incident slit size and as 1/tanθ, and therefore are largely unobservable at high 2θ angles.

**Fig. 8:**
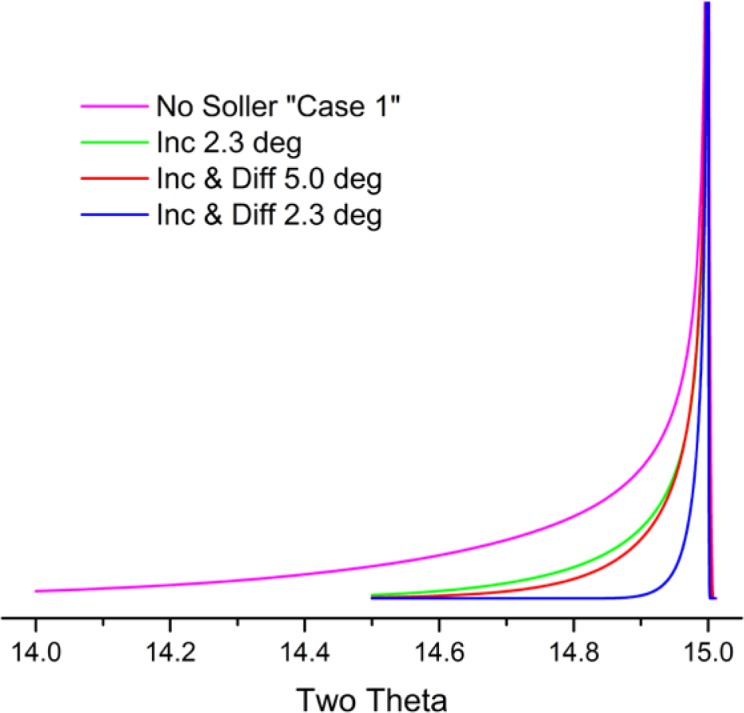
Axial divergence aberration profiles shown for several levels of axial divergence, “Case 1” (of Table 1) is computed for a source length of 12 mm and a sample and receiving slit length of 15 mm. The remaining three simulations include are of “Case 2” wherein Soller slits limit axial divergence, R = 217.5 mm.

**Fig. 9:**
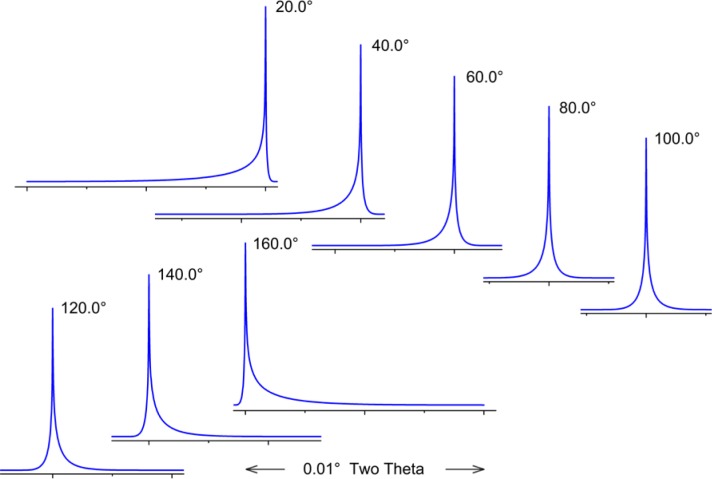
Axial divergence aberration profiles for primary and secondary Soller slits of 2.3° as a function of 2θ, R = 217.5 mm.

**Fig. 10:**
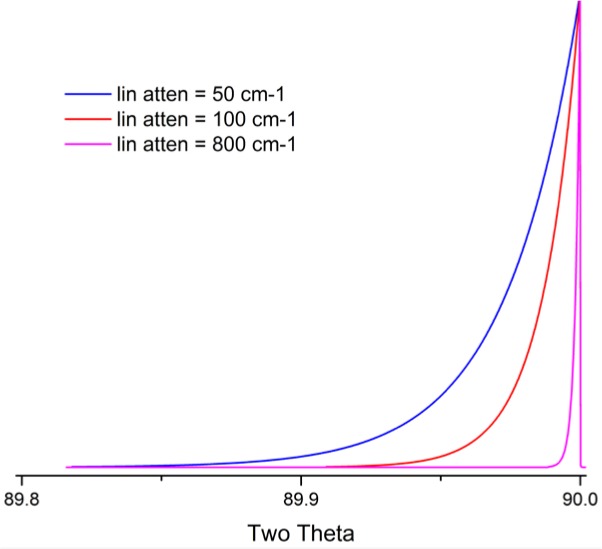
Linear attenuation aberration profiles that would roughly correspond to SRMs 676a (50 cm^−1^), 640e and 1976b (100 cm^−1^) and 660c (800 cm^−1^) at 90° 2θ wherein the transparency effect is at a maximum, R =217.5 mm.

Cheary and Coelho [11, 12] have modeled axial divergence effects in the context of a geometric “Case 1” and “Case 2”. “Case 1” is the situation in which the axial divergence is limited solely by the width of the beam path as determined by the length of the tube filament, the receiving slit and the size of the sample. The aberration function in which these parameters are 12 mm, 15 mm and 15 mm, respectively, is illustrated in Fig. 8; the extent of broadening is nearly one full degree in 2θ, at a 2θ angle of 15°. The other plots of Fig. 8 refer to a “Case 2” situation wherein axial divergence is limited by the inclusion of Soller slits in the incident and diffracted beam paths. One has to consider the impact of a graphite post-monochromator. It will increase the path length of the diffracted beam by 10 cm to 15 cm, reducing axial divergence effects substantially, effectively functioning as a Soller slit. Cheary and Cline [13] determined that the inclusion of a Soller slit with a post-monochromator did result in a slight improvement in resolution; however, this was at the cost of a three-fold reduction in intensity. We do not use Soller slits in the diffracted beam when using a post-monochromator. The 5° primary and secondary Soller slit aberration profile of Fig. 8 corresponds to an instrument with a primary Soller slit and a graphite post-monochromator. The profiles shown for the two 2.3° Soller slits configurations actually constitute a fairly high level of collimation given the double angle definition of the specifications. Shown in Fig. 9 is the functional dependence of the aberration profile, for two 2.3° Soller slits, on 2θ angle. Below approximately 100° 2θ, the effect increases with decreasing 2θ. Approximate symmetry is observed at 100° 2θ, while asymmetry to high angle increases with 2θ thereafter. The aberration profile associated with specimen transparency to the X-ray beam is illustrated in Fig. 10. The figure shows the impact at 90° 2θ wherein the effect is at its maximum. The observed profile is broadened asymmetrically to low 2θ angle; the effect drops off in a largely symmetric manner with 2θ angle on either side of 90°.

**Fig. 11:**
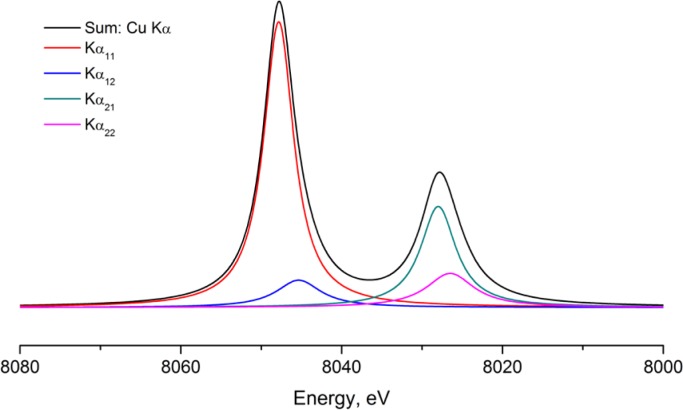
The emission spectrum of Cu Kα radiation as provided by Hölzer, *et al.* (1997), represented by four Lorentzian profiles, two primary ones and a pair of smaller ones to account for the observed asymmetry. The satellite lines, the “Kα_3_” lines, are not displayed.

**Fig. 12:**
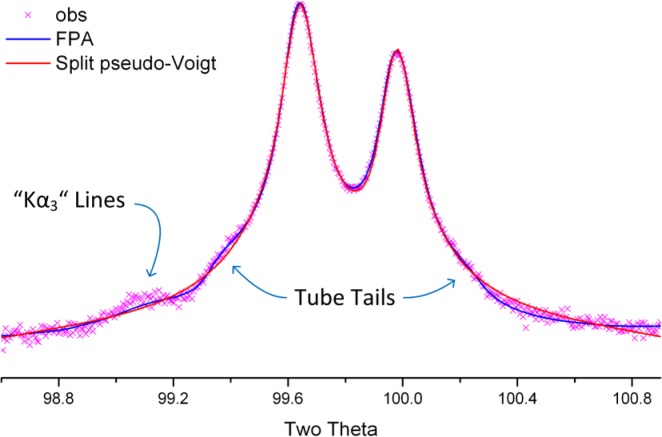
Illustration of the Kα_3_ lines and “Tube Tails” contribution to an observed profile on log scale; shown with two fits: the fundamental parameters approach, which include these features, and the split pseudo-Voigt PSF, which does not.

The wavelength profile or emission spectrum with its characterization on an absolute energy scale provides the traceability of the diffraction measurement to the International System of Units (SI) [14]. The currently accepted characterization of the emission spectrum of Cu Kα radiation is provided by Hölzer *et al.* [15] and is shown in Fig. 11. The spectrum is modeled with four Lorentzian profile shape functions (PSFs): two large ones for the primary Kα_1_ and Kα_2_ profiles, and two smaller ones displaced slightly to lower energy to account for the asymmetry in the observed line shape. The data shown in Fig. 11 are in energy space and are transformed into 2θ space with the dispersion relation. This is obtained by differentiating Bragg’s law to obtain dθ/dλ. The dominant term in the result is tanθ, which leads to the well-known “stretching” of the wavelength distribution with respect to 2θ. Maskil and Deutsch [16] characterized a series of satellite lines in the Cu Kα spectrum with an energy centered around 8080 eV and an intensity relative to the Kα_1_ line of 6 × 10^−3^ These are sometimes referred to as the “Kα_3_” lines, and are typically modeled with a single Lorentzian within the FPA. The “tube tails,” as reported by Bergmann *et al.* [17] are a contribution that is strictly an artifact of how X-rays are produced in the vast majority of laboratory diffractometers. With the operation of an X-ray tube, off-axis electrons are also accelerated into the anode and produce X-rays that originate from positions other than the desired line source. They are not within the expected trajectory of para-focusing X-ray optics and produce “tails” on either side of a line profile as illustrated, along with the Kα_3_ lines in Fig. 12. Lastly, the energy bandpass of the pyrolytic graphite crystals used in post-monochromators is not a “top hat” function. Thus, the inclusion of a post-monochromator influences the observed emission spectrum.

A Johansson IBM dramatically reduces the complexity of the IPF by largely removing the Kα_2_, Kα_3_, and tube tails contributions to the observed profile shape. The vast majority of the Bremsstrahlung is also removed. Furthermore, the inclusion of the IBM increases the path length of the incident beam by 25 cm to 30 cm. This substantially reduces the contribution of axial divergence to the observed profile shape. The crystals used are almost exclusively germanium, the (111) reflection, which are ground and bent to the Johansson focusing geometry, Fig. 3. They can be symmetric, with the ***a***, source-to-crystal distance, and the ***b***, crystal-to-focal point distances being equal, in which case they will exhibit a bandpass on the order of 8 eV. They will “slice” a central portion out of the Kα_1_ line, clipping the “tails”, to transmit perhaps 70 % of the original width of the Cu Kα_1_ emission spectrum. This yields a symmetric profile shape of relatively high resolution, or reduced profile breadth (other parameters being equal). The crystals can also be asymmetric, with the ***a*** distance being ≈ 60 % of the ***b*** distance. These optics will exhibit a bandpass on the order of 15 eV, in which case they transmit the better part of the Kα_1_ line for a higher intensity, but with a lower resolution. The optic discussed herein is of the latter geometry, as shown in Fig. 3.

**Fig. 13:**
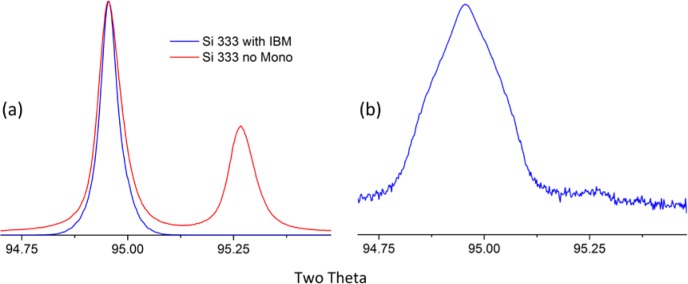
Illustration of the effect of the Johansson optic on the Cu Kα emission spectrum; 13a, data collected on Si 333 single crystal reflection on a linear scale, 13b, analogous data from Johansson optic alone on log scale, both data sets collected with 0.05 mm incident and receiving slits. The near absence of the Kα_2_ scatter displayed in 13b can only be realized with the use of a properly aligned anti-scatter slit located at the focal line of the optic.

A potential drawback to the use of an IBM concerns the nature of the “Kα_1_” emission spectrum it transmits, which may preclude the use of data analysis methods that are based upon an accurate mathematical description of an incident spectrum. At best, a “perfect” focusing crystal will impose an uncharacterized, though somewhat Gaussian, energy filter on the beam it diffracts. However, in certain optics the required bend radius of Johansson geometry is realized by clamping the crystal onto a curved form. The clamping restraint exists only at the edges of the optic, not in the central, active area where it is illuminated by the X-ray beam. The crystal itself however, can minimize internal stress by remaining flat, as such an anticlastic curvature of the optic results. A “saddle” distortion across the surface of the diffracting region of the crystal results in a complex asymmetric “Kα_1_” spectrum that defies accurate mathematical description. Johansson optics, however, can be bent by cementing the crystals into a preform, yielding an optic of superior perfection in curvature. Figure 13 illustrates data collected from such an optic using a Si single crystal, 333 reflection, as an analyzer. Parallel beam conditions were approximated in this experiment with the use of very fine, 0.05 mm incident and receiving slits. The observed symmetric emission profile of Fig. 13a can be modeled with a combination of several Gaussians. However, a Johansson optic will scatter 1 % – 2 % of high energy radiation to a higher 2θ angle than the Kα_1_ focal line of the optic. This undesired scatter is dominated by, but not exclusive to, the Kα_2_ spectrum. Louër (personal communication, 1992) indicated that it can be largely blocked with a knife edge aligned to just “contact” the high-angle side of the optic’s focal line. Alternatively, the NIST method is to use a slit aligned to straddle the focal line. Proper alignment of this anti-scatter slit is critical to realize the level of performance, with the absence of “Kα_2_” scatter, illustrated in Fig. 13b. As will be demonstrated, with use of any Johansson optic the elimination of the Kα_2_ line is of substantial benefit in fitting the observed peaks with analytical profile shape functions.

## 3. Instrument Alignment

Modern instruments embody the drive towards interchangeable, pre-aligned or self-aligning optics, which, in turn, has led to several approaches to obtain proper alignment with a minimum of effort on the part of the user. We will not review these approaches, but instead offer the methods used at NIST with the suggestion that they could be used to review the status of the newer equipment. With the use of calibration methods that simply characterize the performance (errors) of the machine in an empirical manner and apply corrections, the quality of the instrument alignment may be surprisingly uncritical for a number of basic applications such as lattice parameter refinement. However, with the use of the more advanced methods for characterization of the IPF that are based on the use of model functions, the proper alignment of the machine is critical. The models invariably include refineable parameter(s) that characterize the extent to which the given aberration affects the data; the correction is applied, and the results are therefore “correct”. However, in the event of improper instrument alignment, the analysis attempts to model the errors due to misalignment as if they were an expected aberration. The corrections applied are therefore incorrect in degree and nature and an erroneous result is obtained.

**Fig. 14:**
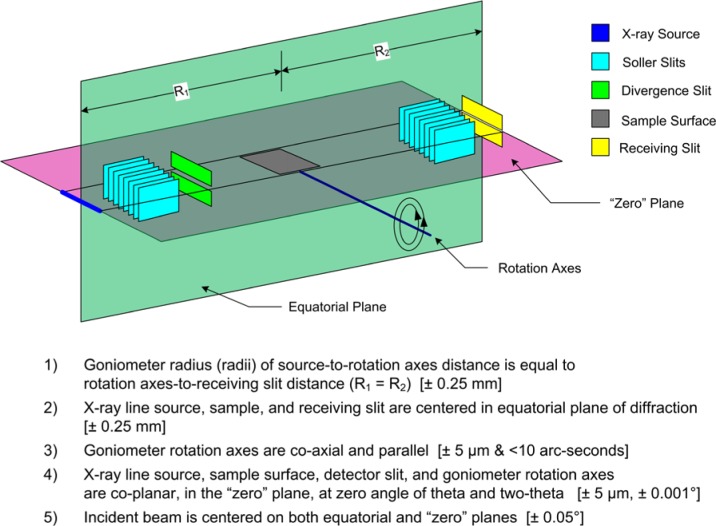
Diagrammatic explanation of the conditions necessary to realize a properly aligned X-ray powder diffractometer.

The conditions for proper alignment of a Bragg-Brentano diffractometer are illustrated and listed in Fig. 14. The first three conditions are considered with the X-rays off, while conditions 4 and 5 are achieved with the beam present as it is actively used in the alignment procedure. Neither incident nor diffracted beam monochromators are considered in Fig. 14; they are simply “added on” to the Bragg-Brentano arrangement and have no effect on the issues outlined therein. Also, to execute the procedure we discuss, a sample stage that can be rotated by 180 degrees in θ angle is required. This need not be the sample stage used for data collection once the alignment procedure is completed, however. Prior to any concerted effort in achieving proper alignment, a straightforward investigation as to the mechanical integrity of the equipment is advisable. Firmly but gently grasp a given component of the diffractometer, such as the tube shield, receiving slit assembly, sample stage, *etc.*, and try to move it in a manner inconsistent with its proper mounting and function. The number of defects, loose bolts, *etc.*, one can find with such an operation, even with equipment with which one is quite familiar, can be surprising.

A brief review of the development of diffraction equipment and the subsequent impact on alignment procedures is appropriate. The goniometer assemblies used for powder diffractometers utilize a worm/ring gear to achieve rotation of the θ and 2θ axes while allowing for the ≈ 0.002 degree resolution with the use of a stepper or servo motor actuating the worm gear. “Home” switches, with a coarse one on the ring gear and a fine one on the worm shaft, allow the software to locate the reference angle(s) of the goniometer assembly to a repeatability of the stepper motor resolution. With the first generation of these automated goniometers, the zero angles were fixed relative to the home positions. With such a design, the invariant reference was the receiving slit, and the operator adjusted the “height” of the tube shield and the angle of the θ stage to realize alignment condition 4 of Fig. 14. Second generation machines offered the ability to set the zero angles relative to the home positions (or those of optical encoders), via software, in which case the exact angular position of either the X-ray tube focal line or of the receiving slit in θ – 2θ space is arbitrary. The operator simply determines the positions where the θ and 2θ angles are zero, and then sets them there. There is no technical reason why the older designs cannot be aligned to the accuracy of newer ones. In practice, however, with the older equipment, the patience of the operator tends to become exhausted, and a lesser result accepted. An important consideration in evaluating modern equipment is that it is often the incident optic, not the X-ray source (focal line), that is used as the reference. Which situation is the case can be readily discerned with an inspection of the hardware: if the incident optic is anchored to the instrument chassis, then it is the reference. If it is attached to the tube shield, however, then the source establishes the reference. The NIST equipment utilizes the latter design.

The first condition listed in Fig. 14 is that the goniometer radius, defined by the source-to-rotation axis distance, R_1_, equals that defined by the rotation axis-to-receiving slit distance, R_2_. This condition is required for proper focusing and is generally realized with the use of rulers to achieve a maximum permissible error of R ± 0.25 mm for a nominal R = 200 mm diffractometer. The second condition concerns the centering of the components in the plane of diffraction or equatorial plane. This condition is assured with the use of straightedges and rulers and, again for a line focus with an 8 mm to 12 mm source length, the maximum permissible error for deviations along the equatorial plane being ± 0.25 mm. One can also consider the takeoff angle at this time; this is the angle between the surface of the X-ray tube anode and the equatorial centerline of the diffractometer incident beam path. As this angle decreases, the resolution is improved at the expense of signal intensity, and vice versa. This is due to variation in the size of the source the specimen “sees”. However, with modern, fine focus tubes, this effect is of second order in nature. Qualitative experiments at NIST indicate that the exact angle is not critical; a 6 degree takeoff angle is reasonable.

The third issue concerns the concentricity of the θ and 2θ rotation axes of the goniometer assembly; this is a matter of underappreciated concern. It is not, however, one in which the end user has a great deal of control. Measurement of axes centricity requires the construction of some fairly complex and stiff structures capable of measuring displacements on the order of 1 µm to 2 μm and rotations of seconds of arc. The objective is to measure both the offset between the two axes and the angle between them. Concentricity errors affect the XRPD data in a manner analogous to that of sample displacement; hence a 5 μm concentricity error is of concern. Worse yet, is the possibility that some degree of precession occurs between the two axes with the operation of the goniometer. In this case the performance of the machine will challenge description using established models.

**Fig. 15:**
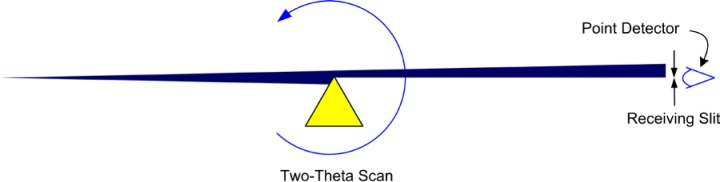
Diagrammatic view illustrating the use of a knife edge to determine the 2θ zero angle.

Subsequent experiments are performed with the X-rays present in order to achieve conditions 4 and 5 of Fig. 14. The criteria for proper alignment illustrated in Fig. 14 are universal in nature. However, there is a range of experimental approaches wherein they can be realized. The specific approach may well be based on the age and manufacturing origin of the equipment as well as the inclinations of the operator. The essence of the experimental design remains constant, however: the operator uses optics mounted in the sample position that will either pass or block the X-ray beam in such a way as to inform the operator if and when the desired alignment condition has been realized. One approach is to use a knife edge mounted as shown in Fig. 15; a 2θ scan is performed using a point detector with a narrow receiving slit. When the intensity reaches 50 % of the maximum, the X-ray source (focal line), the rotation axes of the goniometer and the 2θ (zero) angle are coplanar. However, the problematic presumption here is that the sample stage is aligned with such exactitude that the rotation axes of the goniometer assembly bisect the specimen surface, and therefore the knife edge, to within a few micrometers. This is equivalent to the z height being zero. The verification of this level of accuracy in stage alignment would be exceedingly difficult via direct measurements on the sample stage itself. While many would be inclined to entrust the instrument manufacturer to have correctly aligned the stage, at NIST we use an alternative approach.

**Fig. 16:**
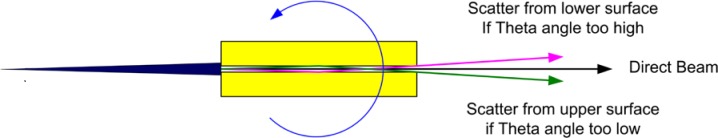
Diagrammatic view of the glass tunnel for determination of θ and 2θ zero angles.

A straightforward means to address this problem is to use a stage that can be inverted, and perform the 2θ zero angle experiment in both orientations. 2θ scans of a knife edge in the normal and inverted positions can be compared to determine the true 2θ zero angle, independent of any z height issue associated with the stage. It is often useful to draw a diagram of the results in order to avoid mix-ups; half the difference between the two measured zero angles yields the true one. With this information, the final alignment involves adjusting the specimen z height in the desired stage, which need not be invertible, until what is known to be the true 2θ zero angle is realized. The knife edge can also be used to center the beam on the rotation axes, as per condition 5. Determination of the θ stage zero angle can be performed using a precision ground flat. An alternative optic to the knife edge is a rectangular “tunnel” with a dimension of 20 μm to 40 μm in width, 10 mm in length, and 5 cm in the direction of the beam path, Fig. 16. Optics of such a nature can be made of metal but are often made of glass. This optic will pass an X-ray beam only if it is parallel to the direction of the tunnel and can be used to determine both θ and 2θ zero angles. These are the optics used at NIST, via an experimental approach that will be discussed subsequently.

If a diffractometer is being commissioned for the first time, or if major components have been replaced, it is appropriate to use fluorescent screens to achieve a rough alignment ensuring that the incident beam does indeed cross the goniometer rotation axes and enter the detector; else one may spend undue time looking for a beam. It is critical that these experiments be performed with the tube at operating power and that the equipment be at thermal equilibrium. Thermal effects will cause the anode to expand and contract, which will typically cause the position of the source to move with it. This is a particularly critical matter when using optics to prepare the incident beam; the performance of which can change markedly with movement of the source.

**Fig. 17:**
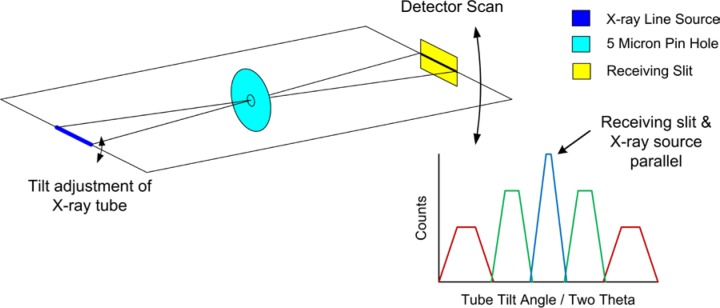
Design and diagram of experiments for using a pinhole optic to align the X-ray source with the receiving slit.

**Fig. 18:**
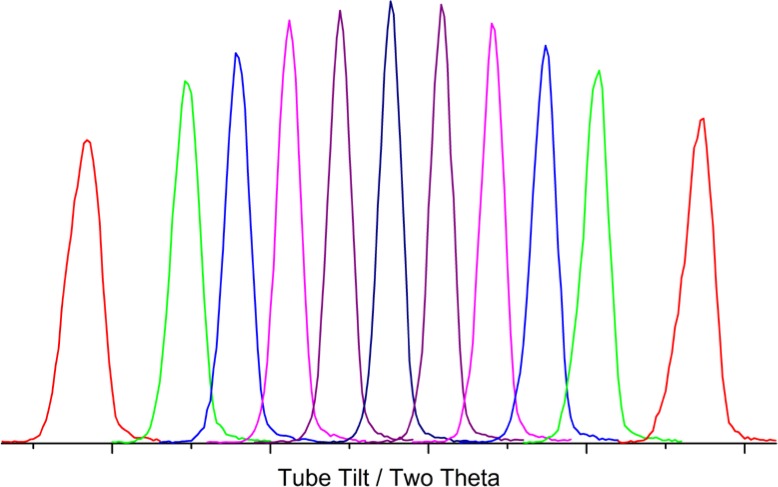
Successful results from the pinhole experiment showing variation in profile shape with successive adjustment of tube tilt; central peak of highest intensity indicates state of parallelism between source and receiving slit.

The object of the first experiment using X-rays is to achieve parallelism between the line source of the tube anode, or focal line of the Johansson optic, and the receiving slit. A 5 µm platinum pinhole, originally manufactured as an aperture for transmission electron microscopy, is mounted in the sample position and used to “image” the focal line of the source onto the receiving slit, Fig. 17. This experiment is the one exception to the “operating power” rule as otherwise Bremsstrahlung will penetrate the platinum foil of the pinhole and provide confounding results. Success can be realized with settings of 20 kV and 10 mA; these reduced power settings are not thought to affect the angle between the tube anode and receiving slit that is the issue addressed in this experiment. The incident slit is opened to the point that the line source itself is imaged, not the incident slit. The Soller slits, and the post-monochromator if so equipped, must also be removed to allow for the axial divergence requisite to the success of this experiment. The pinhole images the line source onto the receiving slit; as the angle between the two decreases, progressively larger lengths of the receiving slit are illuminated during a 2θ scan. The tilt of the X-ray tube shield is varied, and sequential 2θ scans are collected. As parallelism is approached, the profiles will exhibit a progressive increase in the maximum intensity value, with corresponding decreases in breadth. Conclusive results are illustrated in Fig. 18. It should be noted that this is a very difficult experiment to perform because the beam is essentially open and scatter is abundant. Shielding must be installed such that the detector can see only the signal that passes through the pinhole. The pinhole itself should also be shielded to minimize the area of the, relatively transparent, platinum exposed to the direct beam.

**Fig. 19:**
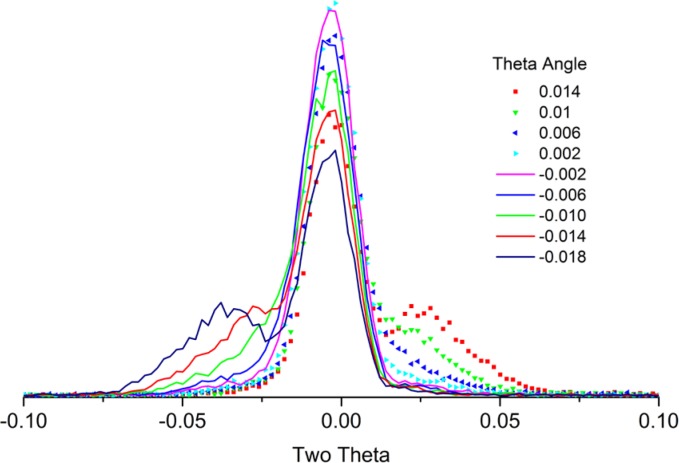
Results from 2θ scans, at successive θ angles using the glass tunnel for determination of θ and 2θ zero angles.

We proceed to determine the θ and 2θ zero angles using the glass tunnel optic. Initial experiments should be performed in the absence of any post-monochromator; its presence tends to complicate finding the beam. However, it should be installed as experiments progress as it will lead to an increase in resolution; it may alter the wavelength distribution slightly and its mass will change the torque moment on the 2θ axis. The latter two factors may alter the apparent 2θ zero by several hundredths of a degree. It is best to use a minimum slit size for the incident beam that will fully illuminate the entrance to the tunnel optic to avoid undue levels of scatter. The receiving slit should be of the smallest size available, 0.05 mm in our case. The first experiment will determine a first approximation of θ zero. The tunnel optic is used with a θ scan being performed with an open detector. Once an approximate θ zero is determined, the receiving slit is installed and a 2θ scan is performed with θ at its zero. Thus, we now have a qualitative idea of both zero angles. Then an experiment is performed as per Fig. 19; sequential 2θ scans are performed as θ is stepped through its zero at exceedingly small steps, 0.004° in the case of our experiment. The tunnel scatters radiation from its upper and lower surfaces when it is not parallel to the central portion of the beam; hence the two lobes on either side of the direct beam of Fig. 19. When θ is at the desired zero angle, the direct beam is transmitted with a minimum intensity in the lobes.

**Fig. 20:**
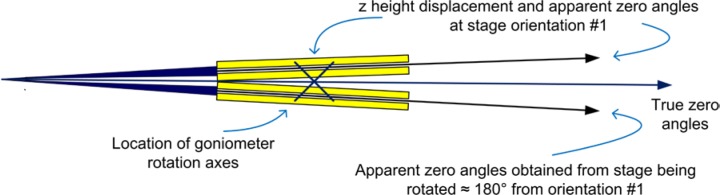
Diagram of hypothetical results from two zero angle measurements (Fig. 19) with the sample stage in the “normal” and “inverted” positions to determine the true 2θ zero angle of the goniometer assembly in the absence of a z height error due to sample stage misalignment.

Once the zero positions of the θ and 2θ angles are determined, the stage is inverted and this set of experiments is repeated. It is desirable to drive the stage by 180°; however, remounting the stage in an inverted position is acceptable if the mounting structure centers the stage to within a few micrometers. Again, it is often useful to draw a diagram of the results from these two zero angle determinations to ensure a correct interpretation of the data; such a diagram is illustrated in Fig. 20. In this example, the sample height is displaced in the positive z direction, else the positions of “orientation #1” and “180° from orientation #1” would be simply reversed. The operator should verify that fully self-consistent results are obtained with respect to the four zero angles shown in Fig. 20. Because the beam is divergent, the difference between the two θ zero angles will not be precisely 180°, as shown in Fig. 20. Again, half the difference between the two measured 2θ zero angles yields the true one, with respect only to the locations of the X-ray source and the goniometer rotation axes. Using the data of Fig. 20 and the goniometer radius, the z height error on the stage in question could be computed and an adjustment made; followed by repeating the two zero angle measurements and checking for self-consistency. This would impart additional confidence in the alignment.

**Fig. 21:**
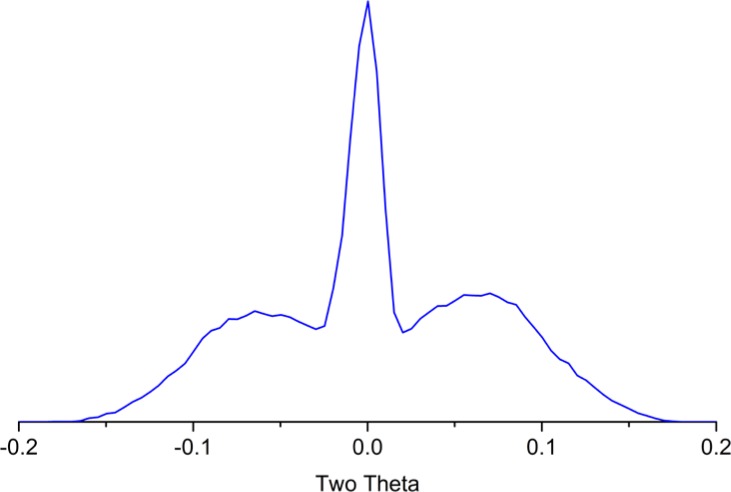
Final results from θ/2θ scan using the glass tunnel indicating correct determination of θ and 2θ zero angles.

The final task is to mount the stage to be used in subsequent data collection and adjust its sample height until the known true 2θ zero angle is obtained. The final experiment is a θ – 2θ scan of the tunnel optic to yield data of the kind shown in Fig. 21. The symmetry of the two lobes on either side of the peak from the direct beam are indicative of the correct θ zero angle setting. This final experiment, of high resolution, is an excellent indicator of the state of the alignment of the instrument. These experiments used in conjunction with profile fitting, can yield measurements of the zero angles with an uncertainty for θ and 2θ of ± 0.001°. Given the high certainty with which the zero angles are determined, they are not refined in subsequent data analyses. The alignment of the incident beam slit, the fifth issue listed in Fig. 14, is accomplished with a scan of the direct beam. If the machine is equipped with a variable divergence incident beam slit, it is important to evaluate it at several settings because changes in the centerline of the beam may occur as the divergence angle is altered. Use of an excessively narrow receiving slit should be avoided for scans of the direct beam, since the thickness of the metal blades used for the slit itself may be larger than the width of the slit, imparting a directional selectivity as the scan is performed.

The work concerning alignment presented here was conducted using a scintillation detector; however, much of it could be performed using a PSD in “picture-taking” mode. In any case, the count rates have to be monitored to ensure that they are within the linear range of the detector, 5K to 10K cps; else anomalous results are obtained. Attenuating foils, flat and in good condition, can be used to reduce intensity. It should also be stressed that, during the experiments, if observations do not adhere to expectations, something is wrong and the desired outcome will not be realized. Diagraming the X-ray beam path can be most useful in discovering the cause of apparently unexplainable observations. Also, throughout these experiments it is appropriate for the operator to try various additional settings to ensure that the machine is operating as expected. Anomalous observations can almost always be explained in a quantitative manner with appropriate investigation. Patience is required.

In the past, achieving acceptable performance with a Johansson optic was considered so problematic that they were underused despite the improvements in the data quality they provided. Modern instrumentation can provide their advantages with dramatically reduced effort. The NIST Johansson IBM, however, was derivative from an older design that was originally supplied with a Siemens D500, *circa* 1987. It uses a Huber 611 monochromator housing that provides 5 degrees of freedom in the positioning of the optic: the ***a*** distance, the takeoff angle, crystal 2θ, tilt and azimuth. For aforementioned reasons, we installed a modern Johansson optic manufactured by Crismatec (now part of Saint Gobain). The procedure for alignment of the machine equipped with the IBM consists of two efforts: first, aligning the crystal optic itself to the line source of the tube anode, and the second, aligning the tube shield/IBM assembly to the goniometer. The latter procedure is analogous to the aforementioned instrument alignment; as such we will discuss only the first issue, though not exhaustively.

**Fig. 22:**
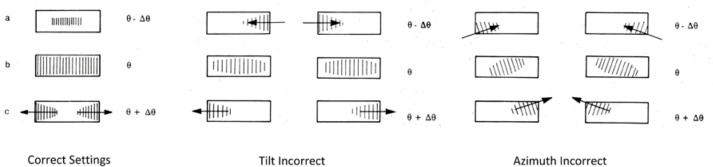
Figures found within instructions for a Siemens D500 incident beam monochromator, Huber 611 monochromator housing, illustrating image formation and movement for correct and incorrect settings of tilt and azimuth angles (reproduced by permission).

The alignment of the Johansson optic to the X-ray source is done largely with the X-rays present. The crystal tilt and azimuth are set through the observation of the diffraction images, observed via a fluorescent screen or camera, from the optic as it is rotated through its diffraction angle. Figure 22, reproduced from the instructions supplied by Siemens, illustrates the manner in which the images form and move, informing the operator of necessary adjustments. Initially, a set of hex drive extensions was obtained that allowed for the remote drive of the optic through its 2θ angle. The source was operated at full power while image movement was observed through a lead impregnated window. Later, a motor drive was installed onto the 2θ actuator of the 611 housing. In the end, the incident beam intensity realized from the optic is dependent upon the operator’s ability to discern the subtleties in the image movement, Fig. 22. Blocking the axially divergent signals from the optic with a high-resolution, 0.05°, Soller slit dramatically improves the sensitivity of this observation on the setting of the tilt and azimuth angles. The inclusion of the Soller slit, however, will reduce the intensity markedly. A complete darkening of the room, including blocking of the shutter lights, as well as allowing time for pupil dilation, can be helpful. However, the use of an X-ray imager or a PSD in “picture-taking” mode improves the quality of the alignment by allowing for a more accurate interpretation of the observations.

The desired outcome is the image forming in the center of the beam path, and splitting symmetrically out to the edges with increasing crystal 2θ angle, Fig. 22. The directions supplied by Siemens and Huber allude to the “fine” adjustment (see Huber [18] for movies) of the tilt and azimuth by examining the structure of the diffracted beam at the optic’s focal point. A fluorescent screen, located at the focal point and set at a 6° angle to the beam path, is used to image the beam structure. With the use of the Soller slit for “coarse” alignment of tilt and azimuth, the desired final image for the “fine” adjustment mode was, indeed, obtained. But it was not possible, even with a deliberate missetting of tilt and azimuth angles, to use the defective images at the focal point as a source of feedback for correction of the settings; they were too diffuse.

The Johansson optic is supplied with ***a*** and ***b*** distances that correspond to the angle of asymmetry in the diffraction planes and the bend radius. The instructions will indicate that an incorrect setting in ***a*** will cause the optic’s diffraction image to move up or down in the plane of diffraction with variation of the crystal 2θ angle. Again, a lack of sensitivity prevents the use of this effect as a feedback loop to set ***a***. Alternative, time consuming, experiments for the optimization of the ***a*** distance of the optic were less than conclusive, and we decided to accept the supplied value for ***a***. As before, we set the takeoff angle at 6 degrees. A critical and quite difficult matter is the alignment of the slit located between the X-ray tube and the crystal optic, not shown in Fig. 3. This slit centers the beam onto the active area of the optic; misalignment leads to unwanted scatter from the optic’s edges. It is aligned with the X-ray beam present, yielding an image of the shadow cast by the optic itself on one side, and one edge of the slit on the other. The optic is rotated in 2θ so that its surface is “parallel” to X-ray beam, *i.e.*, shadowing is minimized. The shadow from the second edge of the slit is obscured by the optic. Geometric considerations are used in conjunction with knowledge of the radius of curvature of the optic to obtain the correct location for the slit. A drawing is highly useful in this instance. After the installation of this slit, it is appropriate to re-check the tilt and azimuth settings as the alignment of the optic is nearly complete.

The setting of the crystal 2θ is performed by evaluation of the direct beam, either with scans using a scintillation detector or by taking pictures with a PSD. With increasing crystal 2θ angle, the beam diffracted by the optic will build in the center forming a broad profile; then the intensity on either side of the initial profile will rise leading to the desired box form; and then intensity at the center of the box will fall, followed lastly by the intensity at either side of the center. This is consistent with the imagery shown in Fig. 22. The process will repeat at half the Kα_1_ intensity for the Kα_2_ line (avoid tuning to the wrong line). The crystal 2θ setting should be checked at regular intervals with a scan of the direct beam; this is the only setting on the IBM observed to drift with time.

The final step in alignment of the IBM is the installation of the anti-scatter slit located at the focal line of the optic, Fig. 3. This is performed after the IBM assembly is aligned to the goniometer. Optimal performance of the anti-scatter slit can be expected only if it is located precisely at the focal line, which itself constitutes the smallest region within which a maximum of X-ray flux is transmitted. Therefore, the NIST alignment procedure includes an experiment using a narrow slit positioned by an x-y translator to evaluate the relative flux of the beam in the vicinity of the focal line. The y direction, Fig. 3, is parallel to ***b*** dimension. A 0.05 mm slit is translated across the beam in the x direction, while intensity readings are recorded from an open detector. This process is repeated for a sequence of y distances. A plot of the recorded intensity vs. x, at the sequence of y settings, will yield a set of profiles which broaden on either side of true ***b***; the narrowest, highest-intensity profile will indicate the location of the focal line. Thus, the experiment determines both the true ***b*** distance and the location, in the x direction, of the focal line. With the knowledge of ***b***, translational adjustment of the IBM assembly may be required to locate the focal line precisely on the goniometer radius. Also, the experiment effectively measures the size of the focal line, in our case, 0.15 mm. A slit of this dimension was fabricated, and the x-y translator was replaced with a standard slit retainer positioned at the desired location. The results, as discussed, are illustrated in Fig. 13.

## 4. SRMs, Instrumentation and Data Collection Procedures

NIST maintains a suite of SRMs suitable for calibration of powder diffraction equipment and measurements, NIST [19]. These SRMs can be divided into various categories based on the characteristic they are most useful for calibrating: line position, line shape, instrument response and quantitative analysis, though some degree of overlap exists. The powder SRMs are certified in batches, typically consisting of several kilograms of feedstock, that are homogenized, riffled and bottled prior to the certification. A representative sample of the bottle population, typically consisting of ten bottles, undergoes certification measurements. The specific size of each lot is based on expected sales rates, mass of material per unit and an expectation of a re-certification interval of 5 to 7 years. When the stocks of a given certification are exhausted, a renewal of the SRM is certified with a suffix letter incremented for each renewal. Hence SRM 640e [20] is the sixth certification of SRM 640, originally certified in 1973. The microstructural character of the SRM artifact and/or the certification procedure itself are expected to change (improve) with each renewal.

To understand the role of an SRM in the calibration of XRPD measurements and equipment, it is helpful to discuss briefly the documentation accompanying an SRM, see also Taylor and Kuyatt [21], “GUM” [22] and “VIM” [23]. NIST SRMs are known internationally as certified reference materials. Accompanying an SRM is a Certificate of Analysis (CoA) which contains both certified and non-certified quantity values and their stated uncertainties. Certified quantity values are determined by NIST to have metrological traceability to a measurement unit - often a direct linkage to the SI. Non-certified values (those lacking the word certified, as presented within a NIST CoA) are defined by NIST as best estimates of the true value provided by NIST where all known or suspected sources of bias have not been fully investigated. Both certified and non-certified quantity values are stated with an accompanying combined expanded (k = 2) uncertainty. Expanded uncertainty is defined as the combined standard uncertainty values for a given certified value multiplied by a coverage factor, k, of 2 and represents a 95 % confidence interval for a given value. The combined standard uncertainties are determined by applying the law of propagation of uncertainty and in common parlance by using the root-sum-of-squares method to combine the sources of uncertainty. The distinguishing characteristic of a NIST-certified quantity value is that all known instrumental measurement uncertainties have been considered, including the uncertainties from the metrological traceability chain. NIST defines uncertainties in two contexts: Type A and Type B. Type A are the random uncertainties determined by statistical methods, for example the standard deviation of a set of measurements. Type B uncertainties are systematic in nature and their extent is usually based on scientific judgment using all relevant information available on possible biases of the experiment. It is the assessment of the technical origin and magnitude of these Type B uncertainties that is a dominant subject of research in the NIST X-ray metrology program.

XRPD SRM-certified quantity values are used primarily for calibration of XRPD measurement systems. The calibration data collected on test instruments also contain the two aforementioned types of errors, random and systematic. It is the systematic measurement errors, or so-called instrument bias, that can be corrected with a calibration. Calibration is a multi-step process: First, certified quantity values are related to the test instrument data. This is done by computing, from these certified quantity values, what would constitute an “ideal” data set from the “measurement method” to be calibrated. The “method” in this case would include the test instrument, its configuration settings and the data analysis method to be used in subsequent measurements. Second, a data set from the SRM is collected and analyzed under the conditions of the “method”. Lastly, with a comparison of the “ideal” data set to the measured one, a calibration curve would be generated. This would establish a correction to the instrument data and yield a calibrated measurement result. For XRPD, this correction has classically taken the form of a calibration function shifting the apparent 2θ indications. There is also the possibility that a comparison of the “ideal” instrument response with the observed one indicates a mechanical, optical or electrical malfunction of the instrument. This, of course, requires further investigation and repair, rather than simply applying a calibration curve.

The generation of a calibration curve as just described can be thought of as a “classical” calibration applicable when the data analysis procedure(s) use empirical methods to parameterize the observations. More recent, advanced methods such as the FPA use model functions that relate the form of the data directly to the characteristics of the diffraction experiment. Parameters of the model describing the experiment are refined in a least-squares context in order to minimize the difference between a computed pattern and the observed one. With the use of methods that use model functions the calibration takes on a different form as the collection and analysis of data can be thought of as replacing the aforementioned multi-step process. The calibration is completed by comparing the results of the refinement with certified quantity values from an appropriately chosen SRM and the known physical parameter values that describe the optical configuration of the test instrument.

Random measurement error, describing the variation of data for a large set of measurements, can be estimated by repeating measurements over an extended period and computing the variance in the data. Furthermore, over time, one could re-calibrate the system and look at the variance of the systematic bias for a given instrument, *i.e.*, the rate of drift in the instrument. One would also have to investigate the sensitivity of both the random error and the variance in the systematic bias as a function of environmental variables such as ambient temperature, power fluctuations, *etc*. This systematic error variance, combined with the prior determined random error variance and the certified value and its uncertainty provides an instrumental measurement uncertainty that can be applied to all measurements from a given instrument. Such an in-field study, however, would take years to complete. Instead, the instrumental measurement uncertainties for a given commercial XRPD measurement system are typically provided by the manufacturer, with the stated caveat that periodic calibrations be performed via factory specifications. The instrumental measurement uncertainties determined through such a study are invariably much larger than those of the NIST-certified quantity values, as they contain both the instrument measurement errors (systematic and random) combined with certified quantity value uncertainties.

NIST maintains a suite of more than a dozen SRMs for powder diffraction; however, one often encounters discussions of non-institutionally-certified standards such as “NAC” (Na_2_Ca_3_Al_2_F_14_), annealed yttrium oxide and silver behenate. Our discussions here principally concern SRMs 640e, silicon, 660b [24] lanthanum hexaboride, 1976b [25] a sintered alumina disc and 676a [26], alumina. SRM 660b has since been renewed as SRM 660c [27]. Most of the work presented herein was performed using SRM 660b; however, SRM 660c could be utilized in any of these applications with identical results. SRMs certified to address the calibration of line position, such as SRMs 640e, 660c and 1976b are certified in an SI traceable manner with respect to lattice parameter. SRM 1976b is also certified with respect to 14 relative intensity values throughout the full 2θ range accessible with Cu Kα radiation. As such, it is used to verify correct operation of a diffractometer with respect to diffraction intensity as a function of 2θ angle, *i.e.*, instrument sensitivity, Jenkins [28], or instrument response. SRM 676a is a quantitative analysis SRM certified with respect to phase purity, Cline *et al.* [29]. While SRM 676a is certified for use as a quantitative analysis SRM, it is also certified with respect to lattice parameters.

Initially with the certification of SRM 640c in the year 2000, the 640(x) SRMs were prepared to minimize sample-induced line broadening. These powders consist of single crystal particles that were annealed after comminution in accordance to van Berkum *et al.* [30]. Their crystallite size distributions (as determined by laser scattering) have a maximum probable size of approximately 4 μm with 10 % of the population being above 8 μm and 10 % of the population below 2.5 μm (with trace quantities below 1 μm). With Cu Kα radiation, silicon has a linear attenuation of 148 cm^−1^, a relatively low value. SRMs 660(x) consist of lanthanum hexaboride, which was prepared to display a minimal level of both size and micro-strain broadening. With the release of SRM 660a, high resolution diffraction using synchrotron radiation must be used to detect microstructural broadening. However, use of lanthanum hexaboride by the neutron diffraction community is problematic as the naturally abundant ^10^B has an extremely high neutron absorption cross section. Lanthanum hexaboride made from ^10^B is essentially opaque to neutrons, rendering it unsuitable for neutron experiments. This problem was addressed with SRMs 660b and 660c by means of a dedicated processing run using a boron carbide precursor enriched with the ^11^B isotope to a nominal 99% concentration. As such, SRMs 660b and 660c are suitable for neutron experiments; they display a miniscule reduction in micro-strain broadening relative to 660a. SRMs 660b and 660c were prepared at the same time using identical procedures and equipment, but in different lots. Lanthanum hexaboride, with Cu Kα radiation, has a linear attenuation of 1125 cm^−1^, a relatively high value. This linear attenuation virtually eliminates the contribution of specimen transparency to the observed data; as such it offers a more accurate assessment of the IPF for a machine of Bragg-Brentano geometry than is available from other SRMs in the suite. The powder of the SRM 660(x) series consists of aggregates, with the crystallite size being approximately 1 μm and the aggregate size distribution being centered at approximately 8 μm for SRM 660a and 10 μm for 660b and 660c. SRM 676a consists of a fine-grained, eqi-axial, high-phase-purity alpha alumina powder that does not display the effects of preferred orientation. It consists of approximately 1.5 μm diameter aggregates with a broad crystallite size distribution centered at 75 nm. As such, the diffraction lines from SRM 676a display a considerable degree of Lorentzian size broadening, with a 1/cosθ dependence.

SRM 1976b consists of a sintered alumina disc; this format eliminates the variable of sample loading procedure from the diffraction data collected from this SRM. The alumina powder precursor for SRMs 1976, 1976a and 1976b consists of a “tabular” alumina that has been calcined to a high temperature, approximately 1500 °C. This calcination results in a phase-pure alpha alumina powder with a plate-like crystal morphology, approximately 10 μm in diameter by 2 µm to 3 μm in thickness, leading to the texture displayed by these SRMs. The feedstock for SRMs 1976, 1976a and 1976b was manufactured with a common processing procedure: The compacts are liquid phase sintered using a 3 % to 5 % anorthite glass matrix; hot forging was used to achieve a compact of approximately 97 % theoretical density. A unique outcome of the hot forging operation used to manufacture these pieces was the axi-symmetric texture imparted to the microstructure. This axi-symmetric nature permits mounting of the sample in any orientation about the surface normal. Furthermore, as the sintered compacts cool, the viscosity of anorthite steadily increases, solidifying at approximately 800 °C. This permits intergranular movement during cooling, at least until 800 °C, and reduces the level of micro-strain that would otherwise build between the grains due to the anisotropic thermal expansion behavior of alumina. However, despite this relaxation mechanism, SRM 1976x still displays a discernable level of Gaussian micro-strain broadening. SRMs 1976a and 1976b were manufactured in a single, custom, production run and as such display a much more uniform level of texture than does SRM 1976. This fact is reflected in the considerably smaller uncertainty bounds on the certified relative intensity values of SRMs 1976a and 1976b compared to the original SRM 1976.

Mounting of powder specimens for analysis using Bragg-Brentano geometry is a non-trivial process that typically requires 20 min to 30 min. The objective is to achieve a maximum in packing density of the powder with a smooth, flat surface. A 5 μm displacement error in the position of the sample surface will have a noticeable impact on the data collected. Side-drifted mounts allow for realization of a flat surface with relative ease, though maximizing the density of the compact can be challenging. Top-mounted specimens can be compacted using a glass plate or bar that allows the operator to see the sample surface through the glass and, in real time, determine the success or failure in obtaining the desired outcome. Some powders, such as that of SRM 640e, “flow” in the mount with the oscillation of the glass plate across the sample surface. Others, such as SRM 676a do not flow at all; but can be “chopped” into the holder and compacted with a single “squish”. Several attempts may be necessary to realize a quality mount. A low-wetting-angle, low-viscosity silicone-based liquid resin, such as those marketed as vacuum leak sealants in high vacuum operations, can be used to infiltrate the compact once mounted; resulting in a stable sample that will survive some degree of rough handling.

**Fig. 23:**
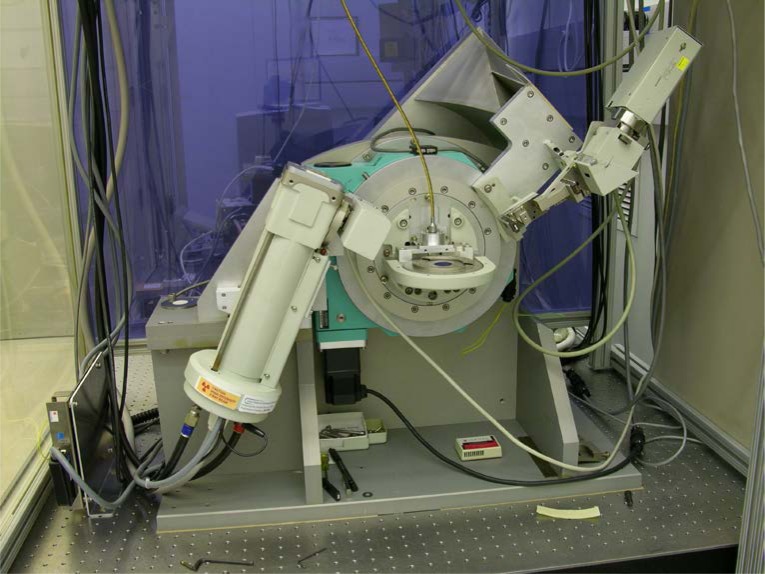
Image of the X ray powder diffractometer, in conventional divergent beam format, that was designed and fabricated at NIST.

**Fig. 24:**
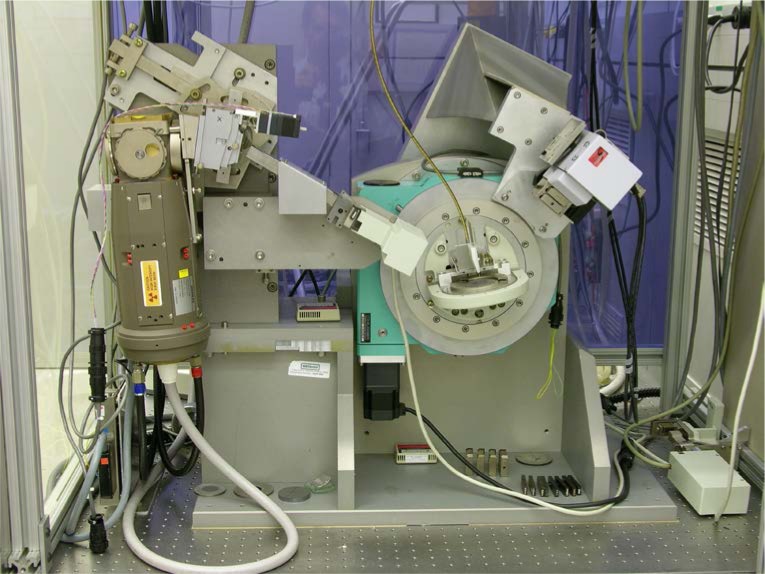
The NIST built powder diffractometer configured with the Johansson incident beam monochromator and position sensitive detector.

The diffractometer discussed in this work is a NIST-built instrument of conventional optical layout, though it is built with several features atypical of equipment of this nature. It was designed and built to produce measurement data of the highest quality. This outcome is not only consistent with the certification of SRMs, but is also requisite to critical evaluation of modern data analysis methods, another goal of this work, which is discussed below. The essence of the instrument is a superior goniometer assembly that is both stiff and accurate in angle measurement, in conjunction with standard, though thoroughly evaluated, optics. The tube shield and incident beam optics are mounted on a removable platform that is located via conical pins that constitute a semi-kinematic mount. This feature allows for a rapid interchange between various optical geometries. Shown in Fig. 23 is the instrument set up in conventional geometry with a post-monochromator and point detector, while Fig. 24 illustrates the setup utilizing a Johansson IBM and PSD. Data from these two configurations are discussed further below.

The goniometer assembly, of θ – 2θ geometry, utilizes a pair of Huber 420 rotation stages mounted concentrically with the rotation axes horizontal. The stage that provides the θ motion faces forward while the 2θ stage faces rearward; they are mounted on a common aluminum monolith, visible in Figs. 23 and 24, which forms the basis of the chassis for the instrument. Both stages incorporate Heidenhain 800 series optical encoders mounted so as to measure the angle of the ring gear. With 4096-fold interpolation provided by IK220 electronics, an angle measurement to within ± 0.00028° (1 arc-second) was realized for both axes. The stages are driven by five-phase stepper motors that incorporate gear reducers of 10:1 for the θ stage and 5:1 for the 2θ, yielding step sizes of 0.0002° and 0.0004°, respectively. The manufacturer’s specifications for the Huber 420 rotation stage claim an eccentricity of less than 3 μm and a wobble of less than 0.0008° (3 arc-seconds). The construction of the goniometer assembly necessitated the development of a specialized jig to align the two 420 rotation stages with regard to both the concentricity (eccentricity) and parallelism (wobble) of their rotation axes. The result was that the overall eccentricity and wobble of the assembly met the specifications cited for the individual stages. The flexing of the detector arm, attached to the rearward facing 2θ stage, was minimized by fabricating a honey-combed aluminum structure, 7.6 cm deep, which maximized stiffness while minimizing weight. Furthermore, the entire detector arm assembly, including the various detectors, was balanced on three axes to minimize off-axis stress on the 2θ rotation stage, Black *et al.* [31]. Thus, the goniometer assembly is exceedingly stiff and offers high-accuracy measurement and control of both the θ and 2θ angles.

The optics, graphite post-monochromator, sample spinner, X-ray generator and tube shield of the machine were originally components of a Siemens D5000 diffractometer, *circa* 1992. As previously discussed, in the IBM configuration, the parts were obtained primarily from a Siemens D500, *circa* 1987. Both configurations include a variable divergence incident beam slit from a D5000. The PSD used in this work was a Bruker Lynxeye XE. The cable attached to the sample spinner, as seen in Figs. 23 and 24, is a flexible drive for the spinner itself; the remote location of the drive motor (not shown) isolates the sample and machinery from the thermal influence of the motor. The machine was positioned on an optical table within a temperature controlled (± 0.1 °C) laboratory space. The temperature of the water used for cooling the X-ray tube and generator was regulated to within ± 0.01 °C. Operation of the machine was provided through control software written in LabVIEW. Data were recorded in true x-y format using the angular measurement data from the optical encoders.

In conventional configuration, the 2.2 kW copper tube of long fine focus geometry was operated at a power of 1.8 kW. This tube gives a source size of nominally 12 mm × 0.04 mm, while the goniometer radius is 217.5 mm. The variable divergence slit was set to ≈ 0.9° for the collection of data discussed here. This results in a beam width, or footprint at the lowest θ angle, on the sample of about 20 mm, conservatively smaller than the sample size of 25 mm. A Soller slit with a divergence of 4.4° defined the axial divergence of the incident beam. A 2 mm anti-scatter slit was placed approximately 113 mm in front of the 0.2 mm (0.05°) receiving slit. The total path length of the scattered radiation, the goniometer radius plus the traverse through the post-monochromator, was approximately 330 mm. This setup reflects what is thought to be a medium resolution diffractometer that would be suitable for a fairly broad range of applications and is therefore a reasonable starting point for a study of instrument calibration. With the IBM, the 1.5 kW copper tube of fine focus geometry was operated at a power of 1.2 kW. This tube gives a source size of nominally 8 mm × 0.04 mm. The variable divergence incident slit was also set to 0.9° with a 0.2 mm (0.05°) receiving slit. The receiving optics were fitted with a 4.4° Soller slit. The total beam path length was ≈ 480 mm.

With the scintillation detector, data were collected using two methods, both of which encompassed the full 2θ range available with these instruments and for which the SRMs offer hkl reflections. The first involves data collection in peak regions only, as illustrated in Table 2 for SRM 660b. The run-time parameters listed in Table 2 reflect the fact that data collection efficiency can be optimized by collecting data in several regions, as both the intensity and breadth vary systematically with respect to 2θ. This was the manner in which data were collected for the certification measurements of SRMs 660c, 640e and 1976b. The second involved a simple continuous scan of fixed step width and count time. It is generally accepted that a step width should be selected to collect a minimum of five data points above the Full-Width-at-Half-Maximum (FWHM) to obtain data of sufficient quality for a Rietveld analysis, Rietveld [32, 33] and McCusker *et al.* [34]. This does not, however, constitute any sort of threshold; collecting data of a finer step width can, with proper data analysis, result in a superior characterization of the IPF. However, one must consider the angular range of acceptance of the receiving slit that is chosen. For a slit of 0.05°, a step width of 0.005° would add only 10 % “new” information; selection of this step width would not constitute a reasonable expenditure of data collection time. We did, however, see fit to collect some data sets we refer to as “ultra-high quality”; the step widths were half, and the count times were approximately 3 times, the values shown in Table 2. For the reported instrument and configuration, the run time parameters of Table 2 result in a minimum of 8 to 10 points above the FWHM. Count times were selected to obtain a uniform number of counts for each profile. It should be noted that it is probably not worth the time to collect quality data from the 222 line of LaB_6_, as it is of low intensity and relatively close to other lines of higher intensity; this is, however, not the case with the 400 line. Selection of the run time parameters can be an iterative process; the total width of each profile scan was set to include at least 0.3° 2θ of apparent background on either side of the profile. Except for the data of SRM 676a, the continuous scans discussed were collected with a step width of 0.008° 2θ and a count time of 4 s to result in a scan time of roughly 24 h. The scans of 676a were collected with 0.01° 2θ step width and 5 s count time.

The PSD used on the NIST diffractometer was a one-dimensional silicon strip detector operated in “picture-taking” mode for all data collection. It has an active window length of 14.4 mm that is divided into 192 strips for a resolution of 75 μm. With a goniometer radius of 217.5 mm this constitutes an active angular range of 3.80° with 0.020° per strip. Slits that would limit the angular range of the PSD window were not used; with each step the counts from all 192 channels were recorded. The PSD was stepped at 0.005° 2θ, for 25 % “new” information per strip; however, to reduce data collection time a second coarse step was also included. Therefore, the data collection algorithm includes the selection of three parameters: a “fine” step (0.005°), the number of fine steps between coarse steps (4), and the size of a coarse step (typically 0.1° or 0.2° 2θ). This approach allows for the collection of high resolution data without stepping through the entire pattern at the high resolution setting. Data presented were collected with four fine steps per detector pixel and a coarse step of 0.1° 2θ. Data were processed to generate the x-y files for subsequent analysis. The operator can select the portion of the 192 channels, centered in the detector window, to be included in the generation of the x-y file. The PSD was fitted with a 1.5° Soller slit for collection of the data presented herein.

## 5. Data Analysis Methods

**Fig. 25:**
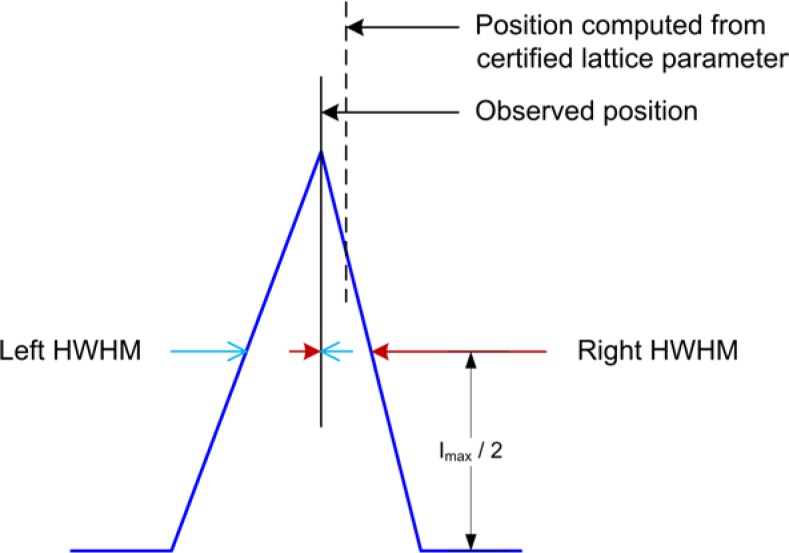
Diagrammatic representation of powder diffraction line profile illustrating delta Two-Theta and HWHM, Half-Width-at-Half-Maximum metrics. The Full-Width-at-Half-Maximum, FWHM, = Left HWHM + Right HWHM.

**Fig. 26:**
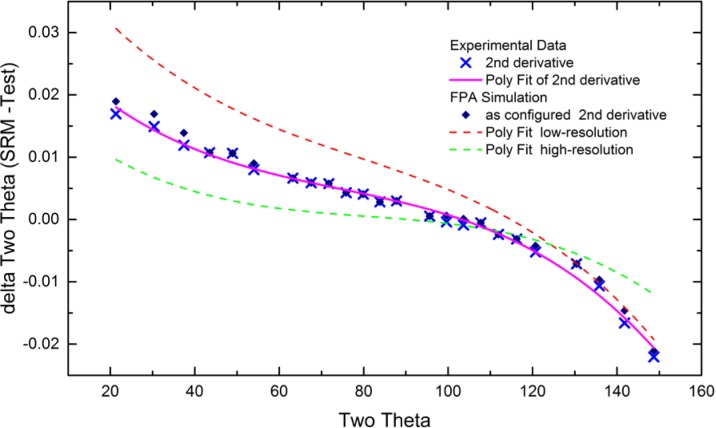
Delta Two Theta curve using SRM 660b illustrating peak position shifts as function of 2θ. Peak positions were determined via a second derivative algorithm, and delta Two Theta values (SRM - Test) were fit with a third-order polynomial. Simulated data are from *FPAPC* and were analyzed via the second derivative algorithm and polynomial fits as per experimental data.

**Fig. 27:**
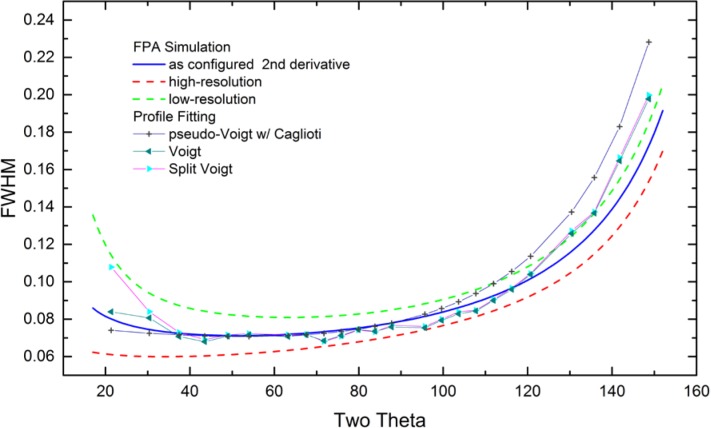
Simulated and actual FWHM data from SRM 660b using the two Voigt PSFs both with and without constraints.

**Fig. 28:**
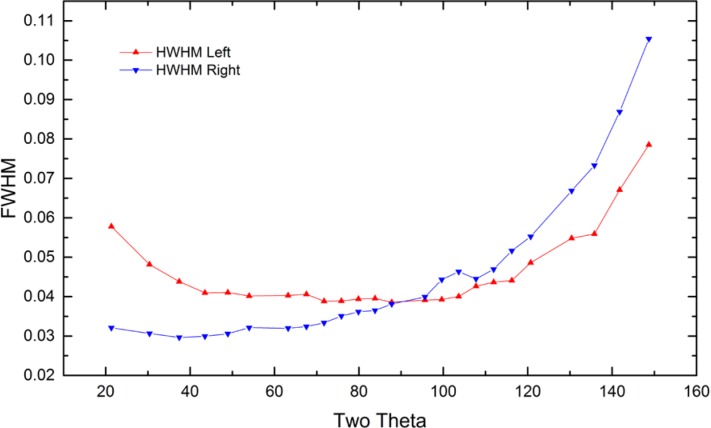
Left and right HWHM data from SRM 660b using the split pseudo Voigt PSF fit with uniform weighting.

As alluded to previously, data analysis procedures can range from the entirely non-physical, using arbitrary analytical functions that have been observed to yield reasonable fits to the observation, to those that exclusively use model functions, derived to specifically represent the effect of some physical aspect of the experiment. The non-physical methods serve to parameterize the performance of the instrument in a descriptive manner. The origins of two of the most common such metrics for consideration of instrument performance are illustrated in Fig. 25. The first is the difference between the apparent position, in 2θ, of the profile maximum and the position of the hkl reflection computed from the certified lattice parameter. These data are plotted *vs.* 2θ to yield a delta Two-Theta curve; a typical example is shown in Fig. 26. An illustration of the Half-Width-at-Half-Maximum (HWHM) defined as the width of either the right or left half of the profile at one half the value of maximum intensity after a background subtraction, is also shown in Fig. 25. These values can be summed, to yield the FWHM, and plotted vs. 2θ to yield an indication of the profile breadth as it varies with 2θ angle, Fig. 27. Additionally the left and right HWHM values of Fig. 28 gauge the variation of profile asymmetry with 2θ; additional parameters of interest, such as the degree of Lorentzian and Gaussian contribution to profile shape, can be plotted *vs.* 2θ to describe the instrument and evaluate its performance.

The least computationally intensive methods for analysis of XRPD data, which have been available since the onset of automated powder diffraction, are based on first- or second-derivative algorithms. These methods report peak positions as the 2θ value at which a local maximum in diffraction intensity is detected in the raw data. Typical software provides “tuning” parameters so that the operation of these algorithms can be optimized for the noise level, step width and profile width of the raw data. These methods are highly mature and offer a quick and reliable means to analyze data in a manner suitable for qualitative analysis and lattice parameter refinement. However, they offer information only as to the position of the peak top. Calibration of the diffractometer via this method is useful only for subsequent analyses that also use such peak location methods.

Profile fitting with an analytical profile shape function offers the potential for greater accuracy because the entire profile is used in the analysis. As with the derivative-based methods, profile fitting also reports the observed 2θ position of maximum intensity, in addition to parameters describing profile shape and breadth. The discussion of the IPF in Sec. 1, as well as a cursory review of Figs. 26 through 28, indicates the complexity in the line profile shape from a Bragg-Brentano instrument. The profiles are symmetric only in a limited region of 2θ; with, in other regions, the degree and direction of profile asymmetry also varying as a function of 2θ. To a first approximation, the optics of an instrument contribute to the Gaussian nature of the profiles; this Gaussian nature will be constant with respect to 2θ. The Lorentzian contribution is primarily from the emission spectrum; given the dominance of angular dispersion effects at high angle, one can expect to see an increase in the Lorentzian character of the profiles with increasing 2θ. While it can be argued that it is physically valid to model specific contributions to the IPF with Gaussian and Lorentzian PSFs, either of these two analytical functions alone cannot be expected to fit the complexities of the IPF and yield useful results. Combinations of these two functions, however, using shape parameters that vary as a function of 2θ, have given credible results for fitting of data from the Bragg-Brentano diffractometer and have been widely incorporated into Rietveld structure refinement software. The Voigt function is a convolution of a Gaussian with a Lorentzian, while the pseudo-Voigt is simply the sum of the two. The parameters that are refined consist of a FWHM and shape parameter that indicates the ratio of the Gaussian to Lorentzian character. The Voigt, being a true convolution, is the more desirable PSF as it is more physically realistic; the pseudo-Voigt tends to be favored as it is less computationally intensive and the differences between the two PSFs have been demonstrated to be minimal, Hastings *et al.* [35], though this is not a matter of universal agreement.

Refining the profile shapes independently invariably leads to errors when analyzing patterns with peak overlap as correlations occur between shape parameters of neighboring profiles. This problem can be addressed by constraining the shape parameters to follow some functional form with respect to 2θ. Caglioti *et al.* [36] developed such a function specifically for constant-wavelength neutron powder diffractometers; it has been incorporated in many Rietveld codes for use with XRPD data. It constrains the FWHM of the Gaussian contribution to the Voigt or pseudo-Voigt PSF:

$${\text{FWHM}}^{2}=U\tan^{2}\theta +V\tan\theta +W,$$ (1)

where the refineable parameters are U, V and W. The term U can be seen to correspond with micro-strain broadening from the sample, and broadening due to the angular dispersion component of the IPF. In *GSAS* an additional term, GP, in 1/cosθ, is included to account for Gaussian size broadening. The Lorentzian FWHM in *GSAS* can vary as:

$$\text{FWHM}=\frac{\text{LX}}{\cos\theta }+\text{LY}\tan\theta ,$$ (2)

where LX and LY are the refineable parameters. Here LX varies with size broadening while LY is the Lorentzian micro-strain and angular dispersion term. Given that the emission spectrum is described with Lorentzian profiles, we would expect the LY term to model the effects of angular dispersion. Within the code *HighScore Plus* the Lorentzian contribution is allowed to vary as:

$$\text{FWHM}=\gamma_{1}+\gamma_{2}2\theta +\gamma_{3}2\theta^{2},$$ (3)

where *γ*_1_, *γ*_2_, and *γ*_3_ are the refineable parameters. Alternatives to the Caglioti function have been proposed that are arguably more appropriate for describing the FWHM data from a Bragg-Brentano instrument, Louër and Langford [37] and Cheary and Cline. However, they have not yet been incorporated into many codes.

The asymmetry in the observed profiles can be fit with the use of a split profile, wherein the two sides of the PSF are refined with independent shape and HWHM parameters. This approach will improve the quality of the fit to the observation; however, it is empirical in nature. The more physically valid approach is the use of models to account for the origins of profile asymmetry. The Finger *et al.* [38] model for axial divergence has been widely implemented in various Rietveld codes. It is formulated to model the axial divergence effects of a synchrotron powder diffraction experiment where the incident beam is essentially parallel. The two refineable parameters, S/L and H/L, refer to the ratios of sample and receiving slit length, relative to the goniometer radius; as such, they define the level of axial divergence in the diffracted beam. This model is not in precise correspondence with the optics of a Bragg-Brentano diffractometer where both the incident and diffracted beams exhibit divergence in the axial direction. It does, however, give quality fits to such data. The use of a model such as this one, as opposed to the sole use of a symmetric or split PSF, will yield peak positions and/or lattice parameters that are “corrected” for the effects of the aberration in question. Therefore, results from the use of model(s) cannot be directly compared with empirical methods that simply characterize the form of the observation. In the case of the Bragg-Brentano experiment, the “correction” that the Finger model applies is not rigorously correct. However, the impact of axial divergence, regardless of the details of diffractometer optics, is universal; as such the use of the Finger model results in a more accurate assessment of “true” peak position and, therefore, lattice parameter.

A third PSF that is in common use is the Pearson VII, or split Pearson VII, that was proposed by Hall *et al.* [39] for fitting of X-ray line profiles. No *a priori* physical justification exists for the use of this PSF. Refineable parameters consist of the FWHM, or HWHM, and an exponent, m. The exponent can range from 1, approximating a Lorentzian PSF, or to ∞ wherein the function tends to a Gaussian. Owing to the lack of a clear physical justification for use of this PSF, it tends to be infrequently employed in Rietveld analysis software.

Convolution-based profile fitting, as diagrammed in Fig. 4, was proposed by Klug and Alexander in 1954 and much of the formalism of the aberration functions shown in Table 1 was developed by Wilson [40]. However, limitations in computing capability largely prevented the realization of the full fundamental parameters approach method until 1992 with the work of Cheary and Coelho. This was made available to the community through the public domain programs *Xfit*, and later *KoalaRiet* [41] and more recently via *TOPAS*. Additional FPA programs are available, most notably *BGMN*, Bergman *et al.* [42]; more recently, *PDXL 2* has had some FPA models incorporated. Within the FPA there are no PSFs other than the Lorentzians used to describe the emission spectrum, the shapes of which are not typically refined. All other aspects of the observation are characterized with the use of model functions that yield parameters descriptive of the experiment. Plausibility of the analysis is determined through evaluation of these parameters with respect to known or expected values. Direct comparison of the results from an FPA to those from methods using analytical PSFs is difficult due to the fundamental difference in the output from the techniques; for example, FWHM values are not obtained directly from the FPA method. However, the NIST *FPAPC* was used to determine FWHM values numerically.

The FPA models of *TOPAS*, *BGMN* and *PDXL 2* were developed specifically for analysis of data from a laboratory diffractometer of Bragg-Brentano geometry. Analyses using this method would be expected to result in the lowest possible residual error terms that characterize the difference between calculation and observation. As has been discussed, the various aberrations affecting the diffraction line shape are such that the observed profile maxima do not necessarily correspond to the d-spacing of the diffracting hkl plane, except perhaps in a limited region of 2θ, emphasizing the need for physically valid modeling of the observed line shape to realize a credible value for the lattice parameter. It being the case that a primary interest of the NIST X-ray metrology program is obtaining said correct value for lattice parameter, our interest in the capabilities of the FPA method is emphasized. Furthermore, experience has demonstrated that the refined parameters obtained through the use of FPA models can be used as in a “feedback loop” to isolate problems and anomalies with the equipment.

Instrument response, or diffracted intensity as a function of 2θ, is considered with a Rietveld analysis by means of models for intensity-sensitive parameters such as crystal structure parameters and Lorentz-polarization factors. The realization of plausible crystal structure parameters from standards via a Rietveld analysis serves as an effective and independently verifiable means to calibrate instrument performance. Consideration of these refined values will offer an effective means of discerning defects that vary smoothly over the full range of 2θ. However, errors observable only within limited regions of 2θ may be difficult to discern with a whole-pattern method; these should be investigated with second-derivative or profile-fitting methods. SRM 676a is used to qualify the machine with respect to instrument response through an ability to reproduce plausible structural parameters and Lorentz-polarization factors. The alumina of SRM 676a is well suited for this purpose because it is non-orienting and of high purity. Alumina is of lower symmetry than either silicon or lanthanum hexaboride; it offers a considerable number of diffraction lines and well-established structure parameters. A Rietveld analysis of SRM 660c, however, yields the IPF in terms of code-specific profile shape terms and verifies that peak position-specific aspects of the equipment and analysis are working correctly.

Instrument response may be evaluated with the more conventional data analysis methods with use of SRM 1976b. Measurements of peak intensities are obtained from the test instrument, typically by profile fitting, and compared with the certified values. However, the use of SRM 1976b for diffraction equipment of differing optical configurations may require that a bias be applied to the certified values to render them appropriate for the machine to be qualified. This bias is needed to account for differences in the polarization effects from the presence, absence, and character of crystal monochromators. The polarization factor for a diffractometer that is not equipped with a monochromator is, from Guinier [43]:

$$\frac{1+\cos^{2}2\theta }{2}.$$ (4)

The polarization factor for a diffractometer equipped with only an incident beam monochromator is, from

$$\frac{1+\cos^{2}2\theta_{m}\cos^{2}2\theta }{1+\cos^{2}2\theta_{m}},$$ (5)

where 2θ_m_ is the 2θ angle of diffraction for the monochromator crystal. The polarization factor for a diffractometer equipped with only a diffracted-beam, post-monochromator is, from Yao and Jinno [45]:

$$\frac{1+\cos^{2}2\theta_{m}\cos^{2}2\theta }{2},$$ (6)

where, again, 2θ_m_ is the 2θ angle of the monochromator crystal. Equations (5) and (6) are considered appropriate when the crystal is of an “ideal mosaic” structure, *i.e*.; the diffracting domains are uniformly small and, therefore, the crystal is diffracting in the kinematic limit. This is in contrast to a “perfect” crystal, which would diffract in accordance with dynamical scattering theory. Note that Eqs. (5) and (6) both have the cos^2^2θ_m_ multiplier operating on the cos^2^2θ term. Since this multiplier is less than unity, machines equipped with a monochromator exhibit a weaker angular dependence.

The certification data for SRM 1976b were collected with the NIST machine equipped with the Johansson IBM and scintillation detector. The simplified IPF of this machine is advantageous for the accurate fitting of the profiles and, therefore, intensity measurement. The validity of this “ideal mosaic” assumption embodied in Eq. (5) was evaluated using this diffractometer; the validity of Eq. (6) was evaluated with data from the machine configured with the post-monochromator. With respect to Eq. (5), for a Ge crystal (111) reflection, 2θ_m_ was set to 27.3°; with regards to Eq. (6), for a pyrolytic graphite crystal (0002) basal plane reflection, 2θ_m_ was set to 26.6°. Rietveld analyses of data from SRMs 660b, 1976b, and 676a, which included a refinement of the polarization factor, modeled as per Eqs. (5) and (6) in *TOPAS*, yielded fits of high quality, indicating that these models were appropriate for these crystals and configurations. Equations (4), (5), and (6) were used to bias the certified values to correspond to those of alternative configurations. These values are included in the SRM 1976b CoA as ancillary data.

## 6. Instrument Calibration

The calibration procedure has traditionally involved the comparison of measurements from a reference, an SRM, with those of the test instrument. However, the exact form of this comparison depends upon the data analyses procedure to be used. A classical calibration, permitting qualitative analyses and lattice parameter refinement, can be readily performed as per Fig. 26. These data are fit with a polynomial that describes the 2θ error correction that is then applied to subsequent unknown samples. Furthermore, with this calibration method, the actual form of the curve of Fig. 26 is largely irrelevant. As the data analysis methods become more advanced, physical models are chosen to replace analytical PSFs. The calibration is then based upon the observation that the machine performance does indeed correspond to the models used, and that acceptable values for refined parameters describing the experiment are obtained from an analysis of data from an SRM. A systematic approach to instrument calibration with a full evaluation of the data, including that obtained from the empirical methods shown in Figs. 26 and 27, results in the ability to use the advanced methods in a rational manner and obtain results in which one can have confidence. The advanced methods, while more complex to use and requiring a much more extensive instrument calibration process, reward the user with a sample characterization of greater breadth and reduced measurement uncertainty.

Consider the delta Two-Theta curve illustrated in Fig. 26. The y-axis values are the difference between the peak positions computed from the certified lattice parameter of SRM 660b, and those of each observed profile determined via a second derivative-based peak location algorithm. Therefore, each of the delta 2θ data points plotted on Fig. 26 were determined independently. It is immediately apparent that the data follow a smooth, monotonic curve with no substantive outliers. Discontinuities or non-monotonicity would typically indicate mechanical difficulties with the equipment, such as loose components or problems with the goniometer assembly. Evaluation of independently determined data such as these are critical to verifying that there are no “high-frequency” difficulties with the equipment that would otherwise be hidden or “smoothed-out” with the use of methods that apply models or constraints across the entire 2θ range, such as a Rietveld analysis. The delta Two-Theta values were fit with a third order polynomial that is also illustrated in Fig. 26. Consideration of the deviation values between the observations and the third order fit indicates a random or “top hat” distribution with a maximum excursion of ± 0.0025° 2θ; this provides further support that a machine is operating properly.

*FPAPC* was used to generate simulated data, which were then analyzed using the same second derivative algorithm as was applied to the raw data. The aforementioned optical setup of the NIST instrument was used in the “as configured” simulation (see Fig. 26 legend); while the “high-resolution” and “low-resolution” data were simulated with a 50 % increase or decrease of the incident and Soller slit angles. For the “high-resolution” and “low resolution” data, third order polynomial fits to the delta 2θ values are displayed in Fig. 26; for the “as configured” data, the delta values themselves are indicated. The correspondence between the simulation and observation indicate that trends in the data can be readily explained in the context of the aberration functions discussed in Sec. l and that such a machine can generate data for successful analysis with the FPA method, *i.e.*, the “metrological loop is closed”. At low 2θ angle the profiles are displaced to low angle by the effects of the flat specimen error and axial divergence. The delta 2θ curve crosses the zero point at approximately 100° 2θ where the profiles are largely symmetric; the slight asymmetry to low angle caused by the flat specimen error is somewhat offset by asymmetry of the emission spectrum to high angle. At higher 2θ angle the profiles are displaced to high angle by the combined effects of axial divergence and the asymmetry of the emission spectrum. As illustrated with the simulations at lower and higher resolution, the experimental curve of Fig. 26 would either “flatten out” or become more “steep”, respectively with changes in instrument resolution. Given the uniformity of the data, and overall plausibility of this delta Two-Theta curve, the third order polynomial fit is used as a reference against which the merits of other techniques can be judged.

It should also be noted that the data and method of Fig. 26 constitute the “low hanging fruit” of powder diffraction. Data analogous to that of Fig. 26 can be used to correct peak positions of unknowns via either the internal or external standard method using a polynomial fit. The external standard method, however, cannot account for specimen displacement or sample transparency effects as can the internal standard method, which is the same procedure applied to a standard admixed with the unknown. Either of these methods will correct for instrumental aberrations regardless of their form; the nature of the curve of Fig. 26 need only be continuous to permit modeling with a low order polynomial. Studies performed in conjunction with the International Centre for Diffraction Data, ICDD, demonstrate that the use of the internal standard method routinely yields results that are accurate to parts in 10^4^, Edmonds *et al*. [46]. Fawcett *et al.* [47] demonstrate the direct relationship between the use of standards, with the vast majority of analyses being performed via the internal or external standard methods, and the number of high quality “star” patterns in the ICDD database. Thus, the community’s collective ability to perform the most routine of XRPD analyses, the qualitative analysis, has been greatly enhanced over the past ≈ 30 years by these most basic methods and the use of SRMs.

The delta Two-Theta and FWHM calibration curves shown in Figs. 27 through 31 were determined via profile fitting, using several PSFs, of the same raw data from SRM 660b used to generate Fig. 26. In general, results from the three commercial codes were in close correspondence. When used on a split PSF, the Caglioti function was applied independently to the left and right FWHM values. A five- to seven-term Chebyshev polynomial was used for modeling the background in these refinements. The “Goodness-of-Fit” (GoF) (also reduced χ^2^ or *R_wp_/R_exp_*) residual error term of the refinements ranged from 1.6 to 1.9, with the unconstrained refinements yielding the slightly improved fits to the data. Figure 32 illustrates the fit quality of typical results using the split pseudo-Voigt PSF. However, as will be demonstrated, the more plausible parameters, particularly in the context of the FWHM values, were often obtained with the more constrained refinements.

**Fig. 29:**
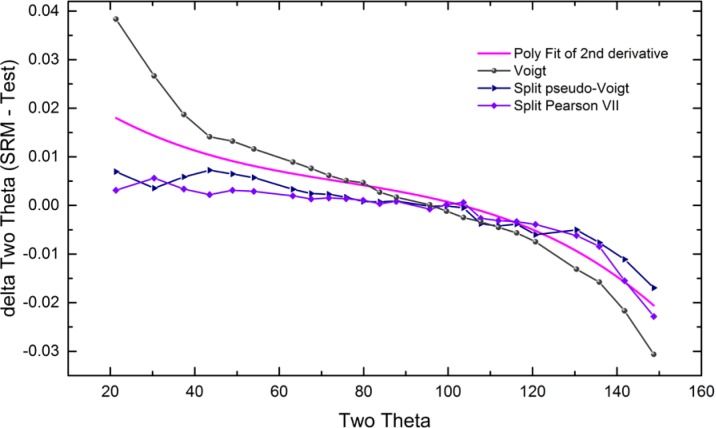
Comparison of Delta Two Theta curves determined with profile fitting of SRM 660b data without the use of any constraints, as a function of 2θ.

**Fig. 30:**
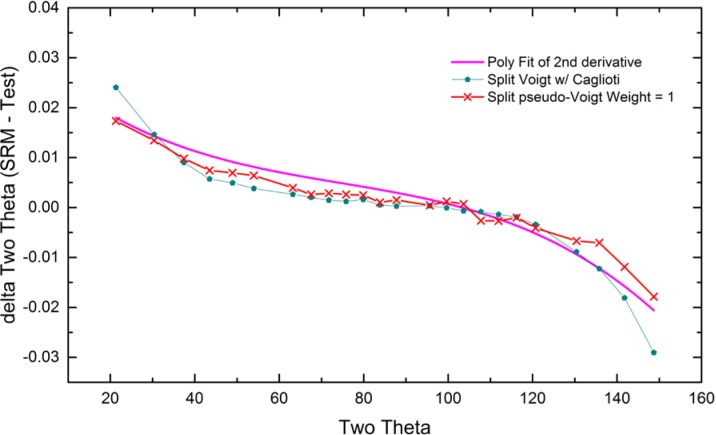
Delta Two Theta curves from SRM 660b determined with profile fitting using the Caglioti function and the and unconstrained split pseudo-Voigt PSF with uniform weighting.

The results from the fitting of Voigt PSF provide a reference for consideration of the delta Two-Theta data of Fig. 29. The use of any of the symmetric PSFs considered herein, with or without the Caglioti constraint, resulted in curves virtually identical to the one displayed in Fig. 29 for the Voigt PSF. Not surprisingly, the symmetric PSF performs quite well in the mid-angle region where the profiles are symmetric but will report an erroneous position in the direction of the asymmetry, when it is present. The opposite effect was observed with the use of any of the split PSFs, however, as can be seen in Figs. 29 and 32. When two HWHM values are refined, the larger HWHM value will “push” the reported peak position in the direction of the smaller one. This effect can be readily observed in the fit quality of the low angle 100 reflection displayed in Fig. 32. The spilt PSFs yield results that reflect an overly asymmetric profile; thus the reported peak positions are displaced to high angle at 2θ angles below 100°, and to low angle at high 2θ angles, above 100°. Curiously this effect was markedly reduced in one of the commercial codes (not shown) and was the sole difference observed between them when the models were equivalent. It is apparent that subtleties in implementation of an ostensibly identical PSF and minimization algorithm (Marquardt) can result in dramatic differences in results. Careful examination of the fit quality is required to access the reliability of profile fitting results. The data of Fig. 29 indicate that errors in peak position of up to 0.015° 2θ are plausible with profile fitting of these data with these PSFs. In contrast to its use with symmetric PSFs, the Caglioti function will improve results when using split PSFs, Fig. 30.

With consideration of the issues related to profile fitting shown in Fig. 32, it was conjectured that fitting the data with a uniform weighting, as opposed to Poisson statistical weighting, *i.e.*, the 1/number-of-counts, may result in more accurate determination of the peak position and FWHM parameters. While this approach would never be used in the vast majority of circumstances because the integrated intensity is a critical metric, it was pursued nonetheless with considerable success. Figure 30 displays data from the use of split pseudo-Voigt that is in very good agreement with second derivative values.

**Fig. 31:**
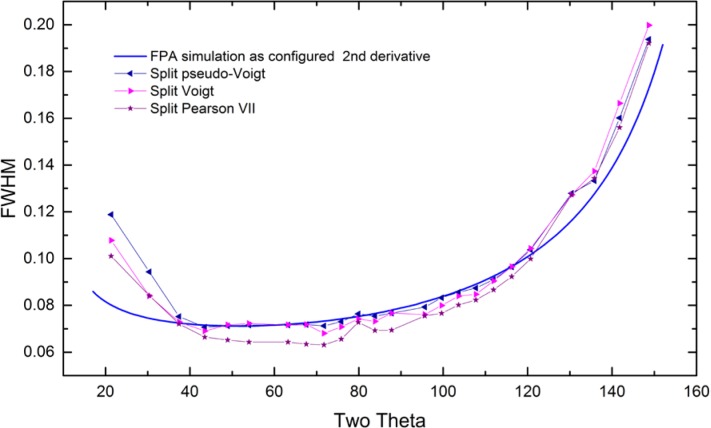
FWHM data from SRM 660b using various split PSFs fitted without constraints.

Experimental and simulated values of FWHM are displayed in Figs. 27 and 31. Data from the profile refinements performed absent the use of the Caglioti function, displayed in Figs. 27 and 31, yield independently determined measures of FWHM. Again, the lack of scatter, and the continuity of these FWHM values are consistent with proper operation of the instrument; *i.e.*, an absence of “high frequency” difficulties. The basic trends are also consistent with the instrument optics: at low 2θ the observed increase in FWHM is due to both the flat specimen and axial divergence aberrations, while at high 2θ, angular dispersion dominates and a substantial increase, with tanθ, in FWHM is apparent. The FPA simulations were performed using the settings for “high” and “low” resolution. The FWHM values were determined numerically from the simulated patterns; no PSF was used. As shown with the simulated data, the degree of upturn at low 2θ increases with a decrease in instrument resolution and *vice versa*. Angular dispersion effects, however, are less dependent on instrument configuration; FWHM values tend towards convergence at high 2θ angle, Fig. 27.

**Fig. 32:**
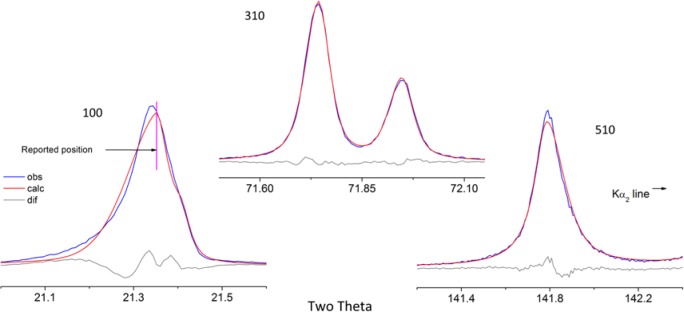
Fits of the split pseudo-Voigt PSF to the low-angle 100, mid-angle 310 and high-angle 510 lines from SRM 660b illustrating erroneous peak position and FWHM reported from 100 and 510 lines, respectively.

As seen in Fig. 27, above 40° 2θ, the Voigt and split Voigt PSFs offer similar values for FWHM, and a fairly accurate representation of instrument performance. It was observed, though not shown, that with regard to the correlation between FWHM values for split vs. symmetric PSFs, the other PSFs behaved in an analogous manner to the Voigt: above 40° 2θ the values reported for FWHM from split vs. symmetric PSFs are nearly identical. From Fig. 31: the split Pearson VII PSF underestimates the FWHM throughout mid-angle region; this error was duplicated with the use of the symmetric Pearson VII PSF (not shown). When fit with uniform weighting, however, these FWHM data from the Pearson VII PSF fell quite precisely (not shown) on the simulated curve. Below 40° 2θ, a split PSF will provide results that overestimate true FWHM values, Figs. 27 and 31. The cause for this is analogous to that discussed for the delta 2θ values, and can be readily observed in the fit quality displayed in Fig. 32 for the low-angle 100 reflection. In accounting for the asymmetry to low angle, the FWHM of the observed profile is substantially overestimated by the calculated one. With all PSFs, the high angle FWHM values are observed to be overestimated, as shown in Figs. 27 and 31; the problem is exacerbated with the use of the Caglioti function. Inspection of the fit quality of the high angle 510 line shown in Fig. 32 indicates that there are two contributions to this effect: One is that the PSF cannot model the shape of the high side of the profile; the other is that the height of the profile is underestimated. These two effects, particularly the inability of the PSF to correctly model the height of the profile, were observed with all of the other PSFs considered here.

The use of the pseudo-Voigt PSF with the Caglioti function results in a reasonable fit to the FWHM values of the observation; the breadth of the high angle lines is overestimated, however. The U, V, and W terms of the Caglioti function vary in a specific manner to account for various physical effects (*e.g.* see Fig. 27): the U term, in tanθ, accounts for angular dispersion; the W term offers the “floor” and the V term accounts for the reduction of the FWHM values in the mid 2θ region. As such the U and W terms should refine to positive values, while the V term should tend to a negative value; negative values for V were, indeed, obtained in these analyses. V should be constrained to negative values or set to zero, as positive values for V are non-physical. With an instrument configured for high resolution, however, values of V = 0 are entirely reasonable as the trend towards an upturn in FWHM at low 2θ angle will be suppressed.

To some extent, the difficulties in determining profile position through the use of these PSFs can be ascribed to the Cu Kα_1_/Kα_2_ doublet as it is “stretched” by angular dispersion. The pattern can be thought of as divided into three regions, each of which will confound fitting procedures in a different manner: the low 2θ range where profiles can be considered as a peak with a shoulder, the mid 2θ range, or perhaps 40° 2θ to 110° 2θ where the profiles can be considered as a doublets, and the high angle region where they are two distinct peaks. This “three-region” consideration is compounded by the direction and severity of the asymmetry in these profiles. The data shown in Fig. 27 largely correspond to the problematic effects of angular dispersion, in the context of these three 2θ regions. These effects are particularly apparent, as shown in Fig. 31, with the use of the Pearson VII function: over-estimation of FWHM values at low 2θ, under estimation in the mid 2θ region, while credible values are obtained at high angle. The use of the Caglioti function is effective in addressing the more extreme excursions from plausible FWHM vales. Figure 28 shows the left and right FWHM values for SRM 660b using the split pseudo-Voigt PSF refined with uniform weighting. For reasons discussed in Sec. 2, the degree, direction, and point of crossover in the profile asymmetry indicated in Fig. 28 are in correspondence with expectation and the previously discussed results from these data from SRM 660b.

**Fig. 33:**
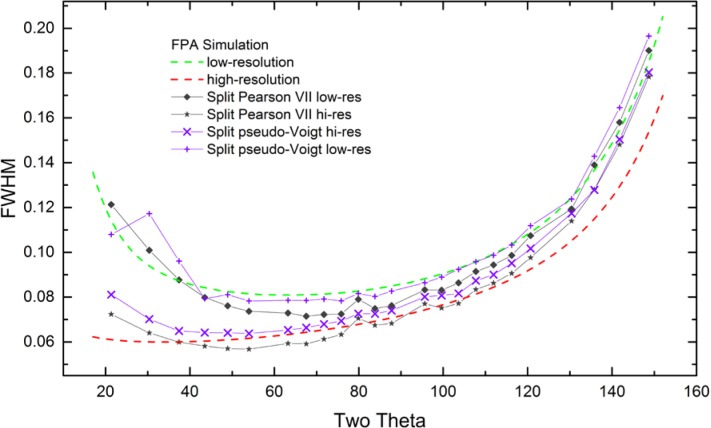
FWHM data from fits of the split pseudo-Voigt and split Pearson VII PSFs to simulated low and high resolution data.

To consider the impact of instrument resolution on the use of analytical PSFs for determination of FWHM values, the simulated high resolution and low resolution data were analyzed via profile fitting. Figure 33 shows the results from the use of the split Pearson VII and spilt pseudo-Voigt PSFs. The data of Fig. 33 indicate an effect that is dependent on the PSF used. The performance of the split Pearson VII PSF is observed to improve with instrument resolution; FWHM values from the narrower profiles are observed to correspond with expectation in the low- and mid-angle regions while substantial deviation is noted with the broader profiles. This is counter to expectation as broader profiles are generally easier to fit than narrow ones. The performance of the split pseudo-Voigt PSF is observed to degrade marginally with either an increase or decrease in instrument resolution. Curiously, the breaths of the high resolution data are overestimated, while those of low resolution data are largely underestimated. Both PSFs do quite poorly in fitting the high angle data from the high resolution setting. These observations emphasize the need to scrutinize the results with an examination of the fit quality, as per Fig. 32.

**Fig. 34:**
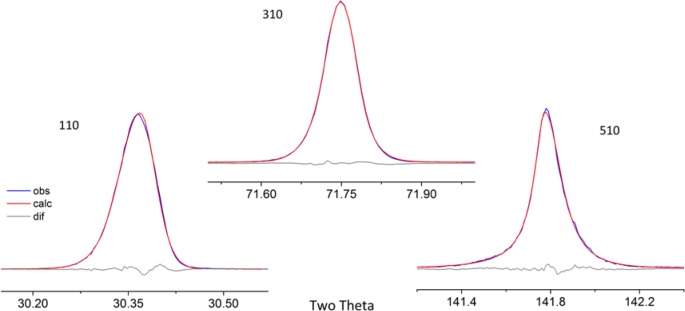
Fits of a split Pearson VII PSF to data from SRM 660b collected using a Johansson IBM.

**Fig. 35:**
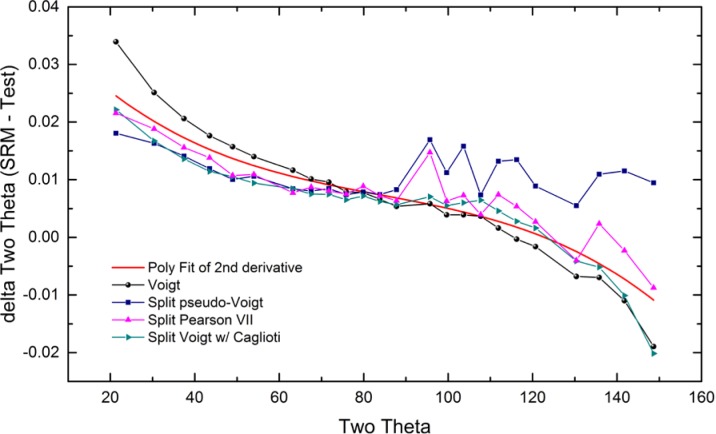
Delta Two Theta curves from the NIST machine configured with a Johansson IBM, illustrating a comparison of results from 2nd derivative and various profile fitting methods. Data are from SRM 660b.

**Fig. 36:**
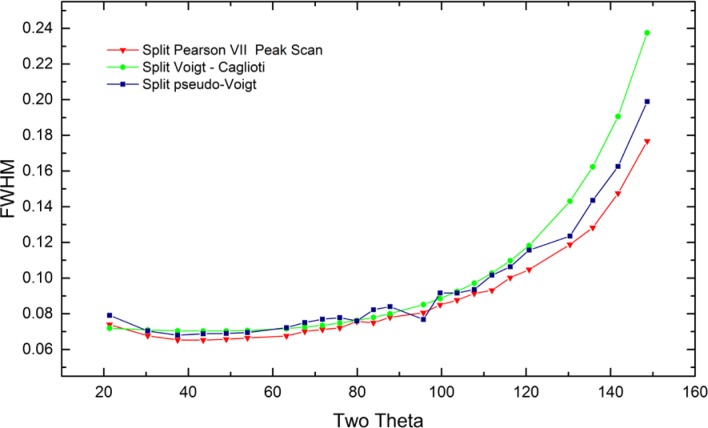
FWHM data from SRM 660b collected on from the NIST machine configured with a Johansson IBM, illustrating a comparison of results from various profile fitting and data collection methods.

When the IPF is simplified with the use of a Johansson IBM, analytical PSFs can provide an excellent fit to the observation, Fig. 34. Illustrated is the fit quality of the split Pearson VII PSF to (high-quality) peak scan data. The split Pearson VII PSF consistently provides a better fit to IBM data than either the split Voigt or split pseudo-Voigt PSFs. Note that the asymmetry exhibited by the profiles follows the same trends as were outlined previously, but to a much reduced extent due to the extended incident beam path length and the resulting reduction in the effects of axial divergence. Figure 35 illustrates the delta Two-Theta calibration curves which were obtained as per the procedures outlined for Fig. 29. Indeed, the trends that are followed, and the reasons why, are largely analogous to those of Fig. 29, but to a much reduced extent due to the reduced profile asymmetry. Use of symmetric PSFs yields reported peak positions that are shifted in the direction of the asymmetry, while use of split PSFs yields positions shifted in the opposite direction owing to the fitted profiles displaying excessive levels of asymmetry. One notes the complete failure of the split pseudo-Voigt, split Voigt (not shown) and, to a lesser extent, the split Pearson VII, PSFs at high angle. However, the more accurate peak positions are obtained from the more intense reflections, indicating that higher quality data may improve results. Improvements in FWHM determination with the use of an IBM are illustrated in Fig. 36 where it can be seen that the pseudo-Voigt and Pearson VII yield values for FWHM that differ in a systematic manner, but to a reduced extent than with the conventional data. The virtues of the peak scan data are illustrated with the lack of “bumps” and “wiggles” in these data relative to the results from the conventional data that were fit with the pseudo-Voigt PSF. The results from the use of the Caglioti function in Fig. 36 illustrate that otherwise noisy FWHM data are effectively “smoothed out,” but a significant bias at high angle is indicated.

**Fig. 37:**
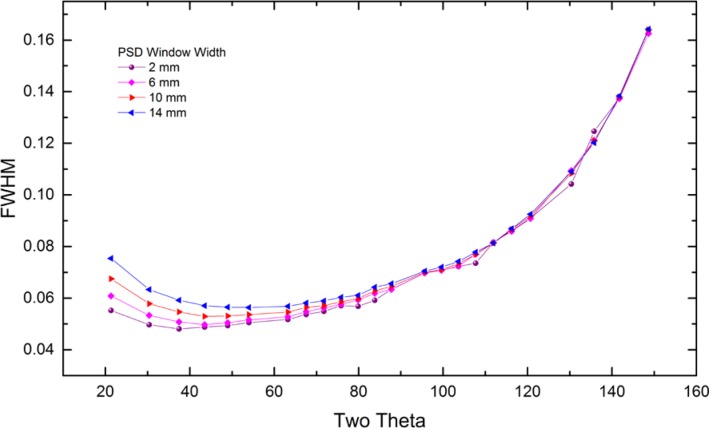
FWHM data from SRM 660b collected on from the NIST machine configured with a Johansson IBM and PSD, illustrating the contribution to defocusing at low angles with increasing window width.

FWHM values from the machine equipped with the IBM and PSD are shown Fig. 37, again with the use of data from SRM 660b. These values were obtained from fits of the split Pearson VII PSF using uniform weighting. The resolution improvement from the use of the PSD is due to the 75 μm strip width, as opposed to the 200 μm receiving slit used with the scintillation detector. This is analogous to a reduction in the “top hat” function used to model the impact of the receiving slit or silicon strip width as discussed in Sec. 2. The impact is greatest at low 2θ angles where the other contributions to overall breadth are small. With increasing 2θ angle, the contribution of a “top hat” function to overall breadth is reduced because it is being convoluted with profiles influenced by ever-increasing spectral dispersion. The improvement in resolution with the reduction in PSD window is apparent, and in conjunction with expectations as per Fig. of Sec. 2. Also, because of the 1/tanθ dependence of this broadening effect, the impact of the window size nearly vanishes above 100° 2θ.

**Fig. 38:**
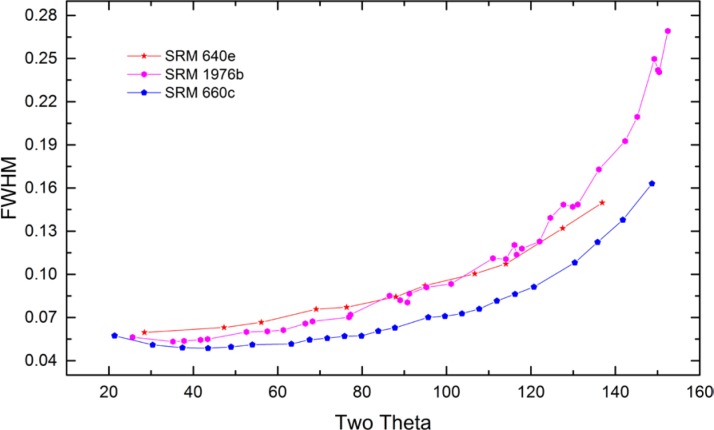
FWHM data from SRMs 640e, 1976b and 660c collected with the IBM and PSD (4 mm window) and fit using the split Pearson VII PSF with uniform weighting.

Figure 38 offers FWHM data obtained for SRMs 640e, 1976b and 660c using the split Pearson VII PSF, fit using uniform weighting on data collected with the IBM and PSD with a 4 mm window. The 660c data set, which exhibits the lowest FWHM values, is discussed first. The FPA analysis performed in the certifications of SRM 660b and 660c included a Lorentzian FWHM with a 1/cosθ dependence to account for size-induced broadening; a domain size of approximately 0.7 µm to 0.8 µm was indicated. There is a high level of uncertainty in these values as they are reflective of an exceedingly small degree of broadening, the detection of which is near the resolution limit of the equipment. The term varying as tanθ, interpreted as micro-strain, refined to zero. These values are found in the CoA for the SRMs. The linear attenuation coefficient for a compact of LaB_6_, with an intrinsic linear attenuation of 1125 cm^−1^ and a particle-packing factor of 60 % to 70 %, would be approximately 800 cm^−1^. Therefore, the contribution to the observed FWHM from specimen transparency with SRM 660c is negligible, as illustrated in Fig. 10. Likewise, the FPA analysis performed for the certification of SRM 640e included size and micro-strain terms; a smaller crystallite size of 0.6 µm was obtained with a very slight amount of micro-strain broadening. However, the linear attenuation coefficient for silicon is 148 cm^−1^; this would lead to approximately 100 cm^−1^ for a powder compact. The transparency of this specimen would lead to significant broadening; see Fig. 10 for the effect of an attenuation of 100 cm^−1^. Therefore, these three effects, in combination, would be expected to lead to a small degree of broadening throughout the 2θ range for SRM 640e, but with a substantial effect in the mid angle region because of the sin2θ dependence of the transparency aberration. Lastly, SRM 1976b is a sintered compact of near theoretical density; therefore, considering the linear attenuation coefficient for alumina, 126 cm^−1^, a value for the actual SRM 1976b specimen of somewhat less than this is expected. An FPA analysis of SRM 1976b indicates a domain size of 1 µm, but with a significant degree of Gaussian micro-strain broadening; this is evident in the observed increase in FWHM with 2θ angle shown in Fig. 38. We conclude that the FWHM data from all three SRMs shown in Fig. 38 are in correspondence with expectations and can be used to select which SRM is best suited for a given application. We do not, however, recommend using an SRM other than SRM 660(x) for a microstructure analysis. It should be added that fitting the profiles of SRM 1976b is complicated by the fact that many of them are overlapping, this leads to the oscillations in the FWHM values shown in Fig. 38 for this SRM. The origins of this difficulty were discussed in Sec. 5 and can be addressed with the use of the Caglioti function.

With the use of model based methods for calibration and subsequent data analysis it is appropriate to consider a strategy for the refinement of the available parameters. The successful refinement will yield the right answer and, with the use of models that make sound physical sense with respect to the experimental design, a good fit to the observation. The refinement strategies for both FPA and Rietveld analyses can be based on a consideration of which terms are specific to the IPF and the manner in which they can be determined. Several parameters can be measured explicitly from experiments other than the diffraction experiment under examination. Examples of these “well determined” parameters include the goniometer zero angles and the incident and receiving slits sizes. Conversely, indeterminate metrics that can only be determined through the diffraction experiment itself include the impact of the post-monochromator on the Cu Kα_1_/Kα_2_ ratio and the degree of axial divergence. Indeterminate parameters specific to the IPF are refined only using high-quality data from standards and fixed for subsequent analyses of unknowns. This approach tends to result in stable and robust refinements. Parameters can, therefore, be considered as falling into three groups: those that are specific to any given sample and are always refined, ones that are specific to the IPF and are refined using only high-quality data from standards, and lastly the highly determined parameters that are refined only as a basic test of the model.

**Fig. 39:**
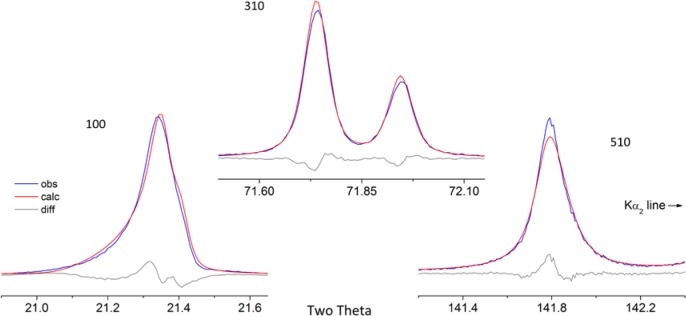
Fits of three SRM 660b lines obtained with a Rietveld analysis using the Thompson, Cox and Hastings formalism of the pseudo-Voigt PSF and the Finger model for asymmetry. Topas was used for the analysis.

To consider the Thompson, Cox and Hastings [48] (TCH) formalism of the pseudo-Voigt PSF with the Finger model for asymmetry, common to many Rietveld codes, a Rietveld analysis of SRM 660b was performed via *GSAS* (using the “type 3” PSF) and *TOPAS* (using “PV_TCHZ” peak type). The TCH formalism allows for the direct refinement of the Gaussian and Lorentzian FWHM values. The Caglioti function was used; Lorentzian terms were constrained as per Eq. (2). The S/L and H/L terms are highly correlated; S/L was refined, while H/L was adjusted manually so that the two terms were nearly equal. Additional parameters that were refined included the lattice parameters, sample displacement and transparency terms, Chebyshev polynomial terms (typically 5 to 7) to represent the background, scale factors, the type “0” Lorentz-polarization term (*GSAS*), the Cu Kα_1_/Kα_2_ ratio, and structural parameters. With this strategy, the sample shift and transparency aberration functions, in conjunction with the Finger asymmetry model, are used to model the data of Fig. 26. Given that the Finger model is not entirely appropriate for divergent beam laboratory data, the sample shift and transparency terms may refine to nonphysical values. They will, however, correctly indicate relative values for sample z height and transparency. The model for specimen transparency in *TOPAS* is the asymmetric function illustrated in Fig. 10, while the model in *GSAS* consists of a profile displacement in sin2θ. The TCH/Finger formalism of *TOPAS* reproduced the certified lattice parameter and resulted in a GoF of 1.5, whereas the GoF value realized with *GSAS* was 1.85. Figure 39 displays the fit quality of the 100, 310 and 510 reflections obtained with *TOPAS*. The fit to the asymmetry of the 100 reflection is reasonable, with a 0.007° shift in position. The fit to the 510 reflection is not dissimilar to that shown in Fig. 32, indicating that the Caglioti function is working analogously to the manner previously discussed. The improvement in fit with the *TOPAS* implementation was most notable around the 70° to 90° 2θ region where the transparency effects are at a maximum. These results validate the TCH/Finger formalism and constitute a valid calibration for this equipment and data analysis method; the utility of the aberration function for specimen transparency as documented by Cheary and Coelho is demonstrated.

Differentiating between the profile shape terms that are specific to the IPF from those refined to consider the microstructure of unknowns yields a stable refinement strategy when using the TCH/Finger formalism. The profile parameters, GU, GV, GW, LX, LY, S/L and H/L so determined from SRM 660b constitute the IPF and are fixed, or used as “floors”, in subsequent refinements, Cline [49]. The IPF for the NIST machine was described with only the GW, LX and LY parameters. In subsequent analyses only the GP, GU, LX and LY terms were refined to represent Gaussian size and micro-strain and Lorentzian size and micro-strain broadening respectively, and thus yield microstructural information from the sample. Parameters that tend to values less than the IPF were fixed at IPF values. The Finger asymmetry parameters determined from the standard need not be refined with unknowns; it has, however, been observed that doing so will neither substantially improve the quality of the fit, nor will it result in instability. Additional parameters that are always refined with unknowns include: scale factors, lattice parameters, specimen displacement and transparency terms, background terms, and structural parameters.

**Fig. 40:**
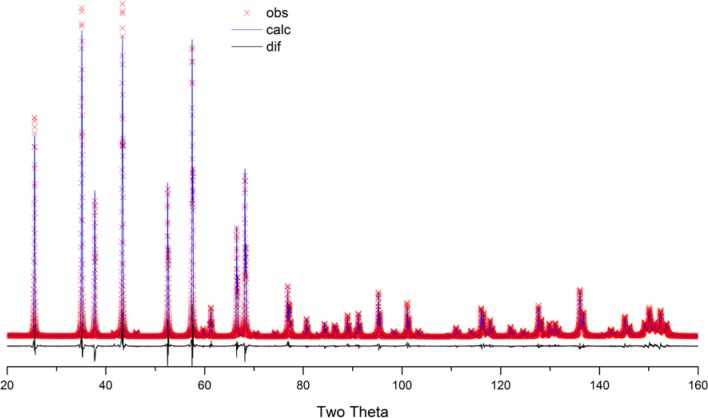
Fits of SRM 676a obtained from a Rietveld analysis using GSAS with the Thompson, Cox and Hastings formalism of the pseudo-Voigt PSF and the Finger model for asymmetry.

While an analysis of SRM 660(x) permits the calibration of the instrument with respect to profile shape and position, it is also desirable to evaluate parameters related to diffraction intensity. However, the analysis of data from high symmetry materials such as silicon and lanthanum hexaboride may result in some degree of instability with the refinement of the intensity-specific parameters, perhaps due to the relatively small number of lines. Use of SRM 676a addresses this difficulty, Fig. 40. With this analysis, the Lorentz-polarization factor refined to a credible value and structure parameters were within the bounds of those obtained from the high q range experiments performed in the certification of SRM 676a, Cline *et al*. (2011).

**Fig. 41:**
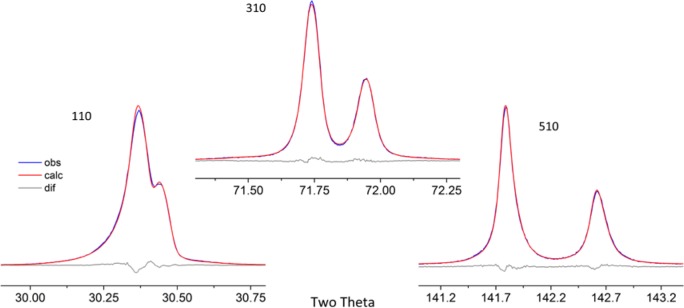
Fit quality realized with a fundamental parameters approach analysis of SRM 660b, peak scan data using Topas.

We initiate the discussion on the FPA method for instrument calibration with a listing of parameters specific to the IPF that would have to be refined with a most basic calibration using an analyses of an SRM. Refined parameters concerning the emission spectrum include the positions and intensities of the Kα_2_ profile, satellite components and the “tube tails”. When addressing the Kα_2_ profile, the relative positions and intensity ratios of the Kα_21_ and Kα_22_ Lorentzian profiles were constrained so as to preserve the overall shape as characterized by Hölzer. With regards to the geometric profile, a parameter characterizing the degree of axial divergence consisting of a single Soller slit value using the “Case 2” axial divergence model applied to both the incident and diffracted beams was refined. Other parameters of the geometric profile were fixed at known values. Additional parameters included a Lorentzian size broadening term, a Gaussian micro-strain term was included for analyses of SRM 1976b, background terms, and profile intensities and positions. Figure 41 illustrates the quality of the fits obtained from an FPA analysis of SRM 660b. These fits present a substantial improvement over those using any of the analytical PSFs, Figs. 32 and 39. Furthermore, the GoF residual error term for an FPA profile analysis of a continuous scan of SRM 660b was 1.08 while the corresponding terms from analyses of the same data using the split pseudo-Voigt and split Pearson VII PSFs were 1.65 and 1.43 respectively (these three analyses all via *TOPAS*). The FPA method can account for subtleties in the observed X-ray line profiles that analytical PSFs could never be expected to fit. In subsequent analyses of unknowns, it is not imprudent to fix parameters associated with the IPF; refining them, however, is typically not problematic with the FPA.

There were indications that the breadths of the Cu Kα emission spectrum as characterized by Hölzer were in excess of those of our observations. This was investigated using the ultra-high quality data. The FWHM ratios of the two pairs of Lorentzian profiles, the Kα_11_ vs. the Kα_12_ and the Kα_21_ vs. the Kα_22_, were constrained to those reported by Hölzer. The positions and intensities of the Kα_2_ doublet were also refined, again with the constraints applied to preserve the shape as per Hölzer. These refinements indicated that the Hölzer breadths were significantly in excess of those that gave the best fit to the data. After an extensive investigation, this observation was confirmed to originate with the performance of the post-monochromator. Several graphite monochromator crystals were investigated using a beam diffracted from a Si single-crystal, 333 reflection, mounted in the specimen position. The graphite crystals that were manufactured within the last 15 years gave an identical result: after an alignment procedure to optimize the intensity of the Kα_1_ line, they do clip the breadths of the emission spectrum by approximately 20 %. They also alter the position of diffraction lines by perhaps 0.01° 2θ; therefore, the goniometer zero angles must be determined with the monochromator installed. We now use the “reduced breadth” Hölzer emission spectrum in our FPA analysis. Note that these breadths vary due to angular dispersion with tanθ, as does micro-strain; therefore, only a “micro-strain-free” specimen can be used for an analysis of the impact of a monochromator on the emission spectrum. We find that both SRMs 660c and 640e are suitable for this analysis.

Another issue that was investigated with the ultra-high quality data concerned the refinement strategy for the “Case 2” Soller slit value. Technically, the axial divergence of the incident beam, with the inclusion of the Soller slit, is less than that of the diffracted beam, which is limited by its extended beam path length through the monochromator. Several strategies were investigated, some of which may have represented a more accurate physical model than that of a single divergence value applied to both beams, but none resulted in any improvement in the fit quality. Lastly, it was observed that the value for the divergence slit, particularly with the use of the IBM, refined to values in excess of the known value. This observation will be discussed further.

With the certification of SRMs 640e, 660c, and 1878b, respirable quartz (2014), global refinements were set up allowing for the simultaneous analysis of the 20 high-quality data sets collected for the certification of the given SRM. With this approach, the analyses could be carried out in the context of highly favorable Poisson counting statistics and permitted a robust analysis of FPA models that would otherwise be problematic due to parameter correlation. Data were collected from two samples from each bottle. With SRMs 640e and 660c, the machine was configured as per the work of this study with the post-monochromator, for 1878b, the machine was configured likewise with the IBM and scintillation detector. For SRM 660c the data were collected in accordance to the run time parameters of Table 2, and in an analogous manner for SRM 640e. For SRM 1878b, the data were collected on mixtures comprised of 50 % SRM 1878b, and 50 % SRM 676a in continuous, 24 h scans. Concurrent with the effort to certify SRMs 640e and 660c, the agreement between the results from *FPAPC* and *Topas* was established indicating that both codes operated in accordance with published FPA models, Mendenhall *et al.* (2015). Initially with *FPAPC* and later with *Topas*, the data from these three SRMs were analyzed using the global refinement strategy.

Several observations concerning possible difficulties with the FPA models were investigated using the global refinements. First, the global refinements were used to determine more robust values for the emission spectrum breadths as influenced by the post-monochromator. The issue concerning the refined value for the incident slit size was revisited with the global refinements. Values of 25 % in excess of the known size were observed in refinements of IBM data from several materials using *TOPAS*. While these refinements were quite robust, corresponding analysis of ultra-high quality post-monochromator data sets resulted in a slow increase in the slit value with little change in residual error terms, indicating a shallow χ^2^ minimization surface. With the global analysis of the SRM 660c, 640e and 1878b data, however, the incident slit value refined to a value of 15 % to 25 % in excess of the known value in a robust manner. The reduced correlations between models with the global refinements lead to this improved ability to realize the minimum in error space for both data types. An investigation into the sensitivity of the lattice parameter value and GoF on the incident slit size was consistent with the shallow χ^2^ minimization surface; changes in lattice parameters were less than 2 fm and only small changes in GoF were noted. The lowest angle lines used in our analyses were at 18°, given the 1/tanθ dependence of the incident slit correction, lower angle lines are required for robust use of this model for refinement of incident slit size. A second observation of concern was low values for sample attenuation refined from data of SRM 660(x). As previously stated, a reasonable value for a compact of LaB_6_ would be 800 cm^−1^, yet the fits were giving values in the 400 cm^−1^ range. Again a sensitivity study indicated little dependence of either lattice parameter or GoF on the attenuation values when they are this large. In contrast, sensitively studies on SRM 640(x), silicon in the 80 cm^−1^ to 100 cm^−1^ range indicated a high level of response to changes in attenuation values. Again, in the range where the model is active, results are in correspondence with expectations; where there is little impact on the refinement, parameters values may differ from true values with little impact on the refinement as a whole. We continue to investigate the issue of the non-physical values obtained for the refined divergence slit value.

**Fig. 42:**
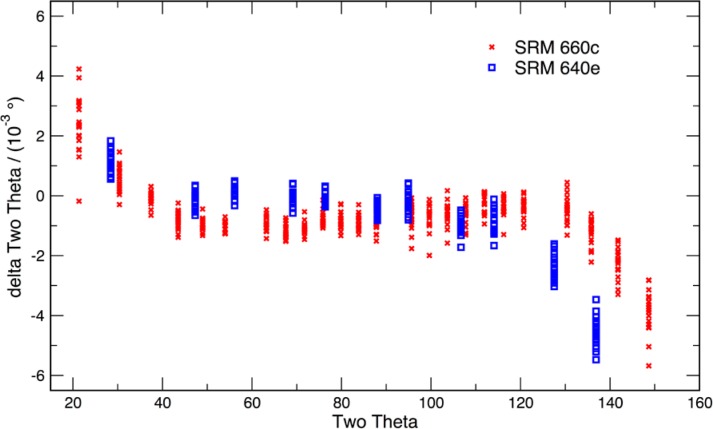
Delta Two Theta data from the twenty data sets collected for the certification of SRMs 660c and 640e, determined via FPA analyses using Topas.

The delta Two-Theta data shown in Fig. 42 illustrates results from an FPA analysis of the twenty data sets collected for the certification of SRMs 660c and 640e. The delta Two-Theta values were generated using the certified lattice parameters of SRMs 660c and 640e to compute “SRM” or reference peak positions, the unconstrained profile positions from the FPA analysis were used as the “Test” data. The analyses were performed using *Topas* with the divergence slit value fixed at the known value. The data of Fig. 42 clearly reflect the efficacy of the FPA method. The certification data for these SRMs were collected on the machine set up as per the data of Fig. 26; the raw trends of the peak position for these data are identical to those of Fig. 26. Yet the FPA has corrected the profile positions to a degree indiscernible from the “true” positions in the 40° to 120° 2θ region. The trends observed otherwise in these data are consistent with prior observations discussed at length in this manuscript; though in 2θ regions limited to below 40° and above 120° and to a vastly reduced level. These deviations are consistent with shortcomings in the model; though the deviations are sufficiently small that it may be problematic to discern their origin. The unequivocal technical justification for use of the FPA in SRM certification is also apparent in Fig. 42, when properly used, the method is capable of reporting the “true” hkl lattice spacing for profiles located in the 40° to 120° 2θ region.

**Fig. 43:**
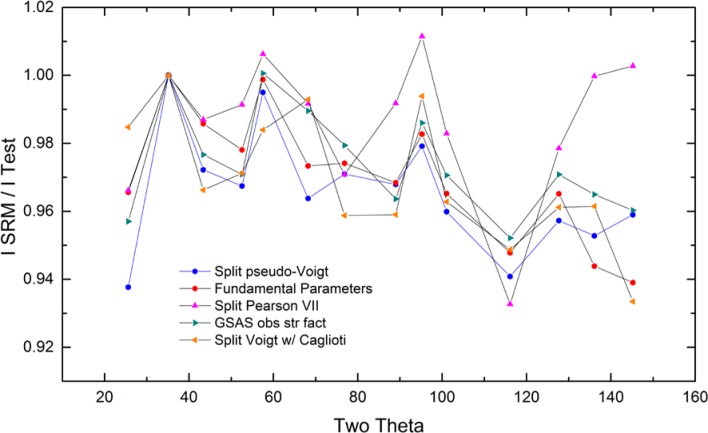
Qualification of a machine using SRM 1976b; data were analyzed using several PSFs.

Employment of SRM 1976b for calibration of instrument response entails determining the integrated intensity of 14 profiles from the test instrument and comparing them with certified values. However, the test instrument in this case was the NIST instrument equipped with the graphite post-monochromator; therefore, the relative intensity values used for comparison were the ones biased to account for the effects of polarization. They were obtained from Table 4 of the SRM 1976b CoA. Figure 43 illustrates the results from various data analysis techniques performed on a common raw data set from the test instrument. With the noteworthy exception of split Pearson VII PSF, all methods gave an acceptable result. It can be seen that when intensity measurement is the issue, the use of unconstrained PSFs is more effective than the previous analyses intended to determine the profile position or FWHM. With the use of *GSAS*, the pattern was fit with a Rietveld analysis using a sixth-order spherical harmonic to model the texture. The reported relative intensity data are computed from the observed structure factors using the *GSAS* utility *REFLIST*. This approach is identical to that used for the certification of SRM 1976b, except the certification data were collected on NIST instrument setup with the IBM.

The structure common to all the data sets of Fig. 43 is as yet unexplained. With any of these methods, modeling the background is of critical concern. The intensity scale of the fitted pattern must be expanded to allow for inspection of the background fit alone. Complicating the matter is the weak amorphous peak associated with the anorthite glass matrix phase observed at approximately 25° 2θ. Certain refinement programs allow for the insertion of a broad peak to account for this, alternatively an 11 to 13 term Chebyshev polynomial was used. Keeping the number of these terms to a minimum is consistent with preventing the background function from interfering with the modeling of the profiles. Lastly, the use of Kβ filters in conjunction with a PSD can be problematic for calibration of instrument response using SRM 1976b. Such filters typically impart an absorption edge in the background on the low energy side of the profiles. With the use of a high-count-rate PSD, this edge can be quite pronounced and cause difficulties in fitting the background and, therefore, erroneous determination of the profile intensity.

## 7. Conclusions

This article reviews the theoretical background behind the well-known complexity of X-ray powder diffraction line profiles. A divergent beam laboratory X-ray diffractometer of conventional layout was constructed in order to rigorously examine the full range of procedures that have been developed for analysis of the instrument profile function. The machine features superlative accuracy in angle measurement with attention paid to precision and stability of optical component and sample positioning. The instrument was aligned in accordance with first principles methods and shown to exhibit optical performance in conformity with the expectations of established theories for powder diffraction optics.

Data analysis methods can be divided into two categories that require fundamentally different approaches to instrument calibration. Empirical profile analysis methods, either based on second derivative algorithms or profile fitting using analytical profile shape functions, seek to characterize instrument performance in terms shape and position parameters that are used in subsequent analysis for determination of specimen character. These methods, however, provide no information as to the origins of the peak shift or profile shape that they describe. Model based methods seek to link the observation directly to the character of the entire experiment. The calibration procedure with the first category can be regarded as a “classical” calibration wherein a correction curve is developed through the use of an SRM and applied to subsequent unknowns. With model based methods, it is beholden to the user to calibrate the instrument in a manner that ensures the models being used correctly correspond to the experiment. This is best accomplished through an analysis of results from empirical methods, particularly the delta Two-Theta curves, as well as an analysis of data from an SRM followed by a critical examination of the refined parameters.

Second derivative-based algorithms for determination of peak location are shown to be competent in providing the 2θ positions, the positions of maximum observed profile intensity, to within ± 0.0025° 2θ. Profile fitting with use of analytical profile shape functions for determination of peak position was shown to be problematic; errors of up to 0.015° 2θ were noted. Use of uniform weighting in the refinements resulted in improved accuracy in the reported peak position and FWHM values. The use of a Johansson incident beam monochromator allows for high quality fits of diffraction data using analytical profile shape functions. The Caglioti function can be used to improve the reliability of FWHM values.

The fundamental parameters approach was found to be effective in modeling the performance of the Bragg-Brentano divergent beam X-ray diffractometer. The form of the delta Two-Theta curve, determined via a second derivative algorithm, was explained quantitatively through an examination of FPA models. Furthermore, FPA simulations of diffraction data, computed from the instrument configuration using both commercial and NIST FPA codes, and analyzed using the same second derivative algorithm, reproduced the delta Two-Theta results from the experimental data. This self-consistency verified the correct operation of both the instrument and the FPA models. The use of the FPA for modeling of the diffraction profiles provided the best fits to the observations and the most accurate results for the “true” reported peaks positions. The TCH/Finger models for profile shape yield credible results for refinement of lattice parameters via the Rietveld method.

## Figures and Tables

**Table 1:** Listing of the aberrations comprising the geometric component of the IPF

Aberration	Controlling parameters	Impact
X-ray Source Width (w_x_)	Angle subtended by source: $\frac{w_{x}}{R}$	Symmetric broadening
Receiving Slit Width or PSD Strip Width (w_r_)	Angle subtended by slit/strip: $\frac{w_{r}}{R}$	Symmetric broadening
Flat Specimen Error/Equatorial Divergence	Angle of divergence slit: α	Asymmetric broadening to low 2θ, with decreasing 2θ
PSD Defocusing	PSD window width, Angle of divergence slit: α	Symmetric broadening with 1/tan(θ)
Axial Divergence Case 1: No Soller slits Case 2: Soller slits define divergence angle	Axial lengths of the x-ray source (L_x_) sample (L_s_) and receiving slit (L_r_) relative to goniometer radius (R) Acceptance angles Δ_I_ and Δ_D_ of the incident and diffracted beam Soller slits	Below ≈ 110°: Asymmetric broadening to low 2θ, with decreasing 2θ Else to high 2θ, with increasing 2θ
Specimen transparency	1 Penetration factor relative to diffractometer radius $\frac{1}{\mu R}$	Asymmetric broadening to low 2θ, with Sin(2θ)
Specimen Displacement Z height	Displacement of specimen surface from goniometer rotation axes	Displacement of profiles with Cos(θ)

**Table 2:** Run time parameters used for collection of data used for certification of SRM 660b; included is the “overhead time” associated with the operation of the goniometer

hkl	Start angle	End angle	Step width (°)	Count time (s)	Total peak time (min)
100	20.3	22.2	0.01	2	6.3
110	29.1	31.4	0.01	1	3.8
111	36.4	38.4	0.01	3	10.0
200	42.7	44.4	0.01	5	14.2
210	48	50	0.008	2	8.3
211	53.2	54.896	0.008	5	17.7
110	62.5	64.204	0.008	11	39.0
300	66.7	68.596	0.008	4	15.8
310	70.9	72.7	0.008	6	22.5
311	75	76.904	0.008	9	35.7
222	79.3	80.804	0.008	47	147.3
320	83	84.904	0.008	15	59.5
321	86.9	88.9	0.008	8	33.3
400	95	96.704	0.008	42	149.1
410	98.6	100.8	0.008	9	41.3
330	102.7	104.9	0.008	12	55.0
331	106.9	108.9	0.01	27	90.0
420	111.1	113.1	0.01	20	66.7
421	115.3	117.6	0.01	10	38.3
332	119.9	122.1	0.01	19	69.7
422	129.6	131.796	0.012	32	97.6
500	134.9	137.396	0.012	27	93.6
510	140.5	144	0.014	7	29.2
511	147.5	150.908	0.016	15	53.2
				**Total time, hrs**	20.0

## Footnotes

1 Certain commercial equipment, instruments, or materials are identified in this in order to adequately specify the experimental procedure. Such identification does not imply recommendation or endorsement by the National Institute of Standards and Technology, nor does it imply that the materials or equipment identified are necessarily the best available for the purpose.
